# Role of neutrophil extracellular traps in regulation of lung cancer invasion and metastasis: Structural insights from a computational model

**DOI:** 10.1371/journal.pcbi.1008257

**Published:** 2021-02-17

**Authors:** Junho Lee, Donggu Lee, Sean Lawler, Yangjin Kim

**Affiliations:** 1 Department of Mathematics, Konkuk University, Seoul, Republic of Korea; 2 Mathematical Biosciences Institute, Ohio State University, Columbus, Ohio, United States of America; 3 Department of neurosurgery, Brigham and Women’s Hospital & Harvard Medical School, Boston, Massachusetts, United States of America; University of Southern California, UNITED STATES

## Abstract

Lung cancer is one of the leading causes of cancer-related deaths worldwide and is characterized by hijacking immune system for active growth and aggressive metastasis. Neutrophils, which in their original form should establish immune activities to the tumor as a first line of defense, are undermined by tumor cells to promote tumor invasion in several ways. In this study, we investigate the mutual interactions between the tumor cells and the neutrophils that facilitate tumor invasion by developing a mathematical model that involves taxis-reaction-diffusion equations for the critical components in the interaction. These include the densities of tumor and neutrophils, and the concentrations of signaling molecules and structure such as neutrophil extracellular traps (NETs). We apply the mathematical model to a Boyden invasion assay used in the experiments to demonstrate that the tumor-associated neutrophils can enhance tumor cell invasion by secreting the neutrophil elastase. We show that the model can both reproduce the major experimental observation on NET-mediated cancer invasion and make several important predictions to guide future experiments with the goal of the development of new anti-tumor strategies. Moreover, using this model, we investigate the fundamental mechanism of NET-mediated invasion of cancer cells and the impact of internal and external heterogeneity on the migration patterning of tumour cells and their response to different treatment schedules.

## Author summary

When cancer patients are diagnosed with tumours at a primary site, the cancer cells are often found in the blood or already metastasized to the secondary sites in other organs. These metastatic cancer cells are more resistant to major anti-cancer therapies, and lead to the low survival probability. Until recently, the role of neutrophils, specifically tumor-associated neutrophils as a member of complex tumor microenvironment, has been ignored for a long time due to technical difficulties in tumor biology but these neutrophils are emerging as an important player in regulation of tumor invasion and metastasis. The mutual interaction between a tumor and neutrophils from bone marrow or in blood induces the critical transition of the naive form, called the N1 type, to the more aggressive phenotype, called the N2 TANs, which then promotes tumor invasion. In this article, we investigate how stimulated neutrophils with different N1 and N2 landscapes shape the metastatic potential of the lung cancers. Our simulation framework is designed for boyden invasion chamber in experiments and based on a mathematical model that describes how tumor cells interact with neutrophils and N2 TANs can promote tumor cell invasion. We demonstrate that the efficacy of anti-tumor (anti-invasion) drugs depend on this critical communication and N1→N2 landscapes of stimulated neutrophils.

## Introduction

Lung cancer is still the leading cause of cancer-associated deaths worldwide, with 1.8 million deaths in 2018 [1, 2]. Various cell types such as immune cells, fibroblasts, and endothelial cells in a tumor microenvironment (TME) interact with tumor cells via the cytokines and growth factors. Tumor-associated neutrophils (TANs) are of particular interest because experimental studies showed that they can contribute to the tumor growth, critical invasion, epithelial-mesenchymal transition (EMT), and metastasis of cancer cells [3, 4]. Until recently, neutrophils have been considered as merely a bystander in the TME and metastasis [5–7] but they are emerging as an important player due to consistent and continuous evidences of their tumor-promoting roles [3]. It was shown that cancer cells can secrete CXC chemokines, one of four main subfamilies of chemokines, attracting neutrophils to tumor microenvironment [8] and neutrophil invasion is highly correlated with poor clinical outcomes [9, 10]. While the classical form of neutrophils, called N1 TANs, can effectively eliminate tumor cells via lysis [11–13], TNF-*α* [14], or inducing tumor cell apoptosis [15], another form, called N2 TANs, can support tumor growth, invasion, metastasis [16–20] and ultimately, poor clinical outcomes in many cancers [21]. Metastatic cancer cells were also able to induce neutrophils to form metastasis-promoting NETs without involving infection processes [22].

**Fig 1:**
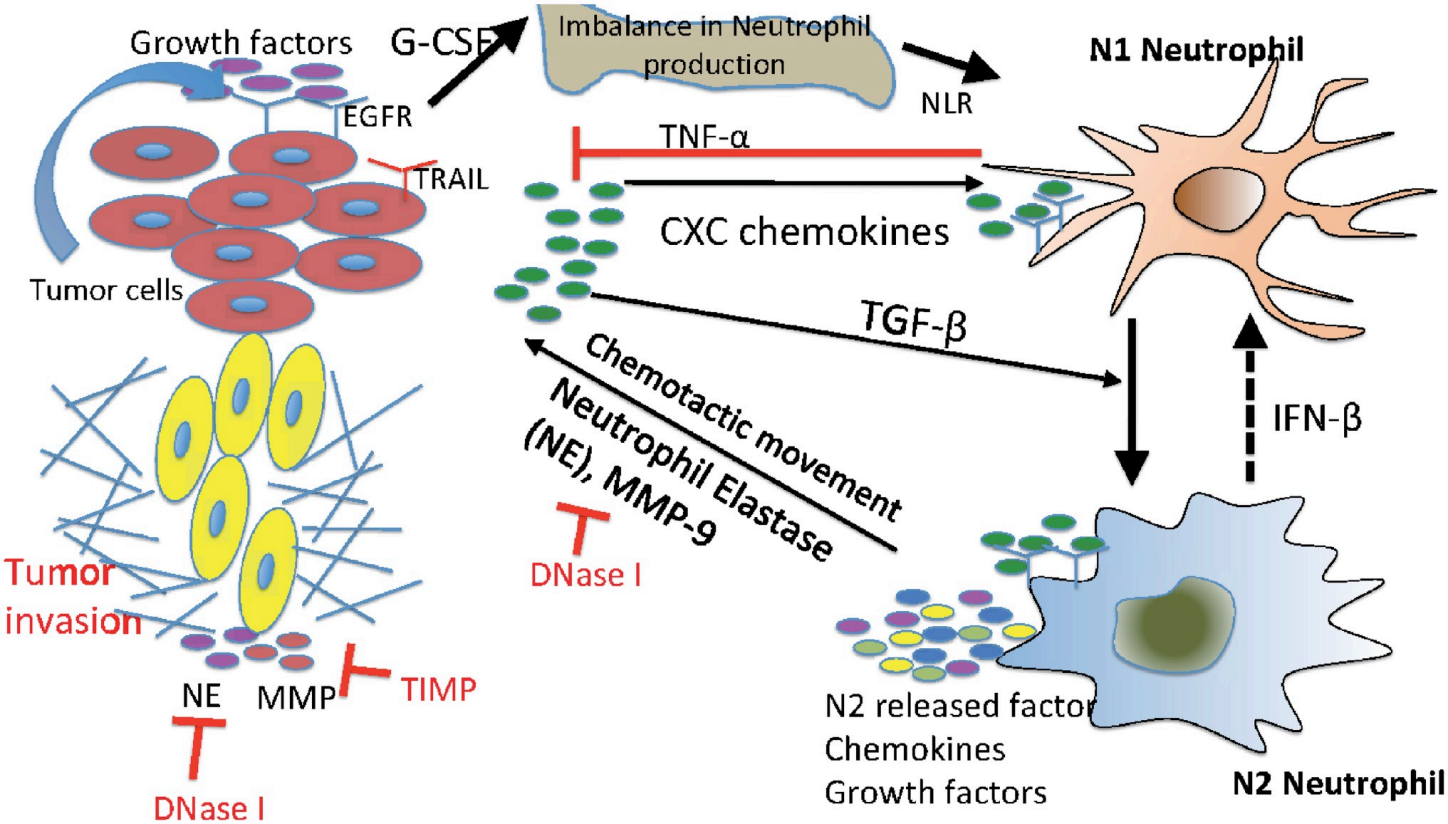
Interaction of the TGF-*β*, IFN-*β*, and NE-pathways in the control of tumor cell invasion. In homeostasis of normal tissue, these pathways are balanced so as to control growth, but in lung cancer, increased secretion of TGF-*β* by tumor cells induces the N1→N2 transition of the neutrophils and stimulates their secretion of NE and other growth factors. This disrupts the homeostasis and stimulates aggressive tumor invasion.

While the tumor-secreted transforming growth factor (TGF-*β*) was shown to transform N1 TANs (tumor-suppressive phenotype) to N2 TANs (tumor-promoting phenotype) [23–25], the N2→N1 transition can be mediated by type I IFN [14, 23, 26, 27] (Fig 1). Neutrophil elastase (NE or ELANE) as well as matrix metallopeptidase (MMP) was shown to infiltrate the TME [28] and promote tumor growth and invasion of cancer cells through the PIK3 signaling pathways [8, 29, 30]. More importantly, it was shown that neutrophils can promote the tumor cell invasion in the transwell assay [22, 31] and *in vivo* experiments [22, 32].

**Fig 2:**
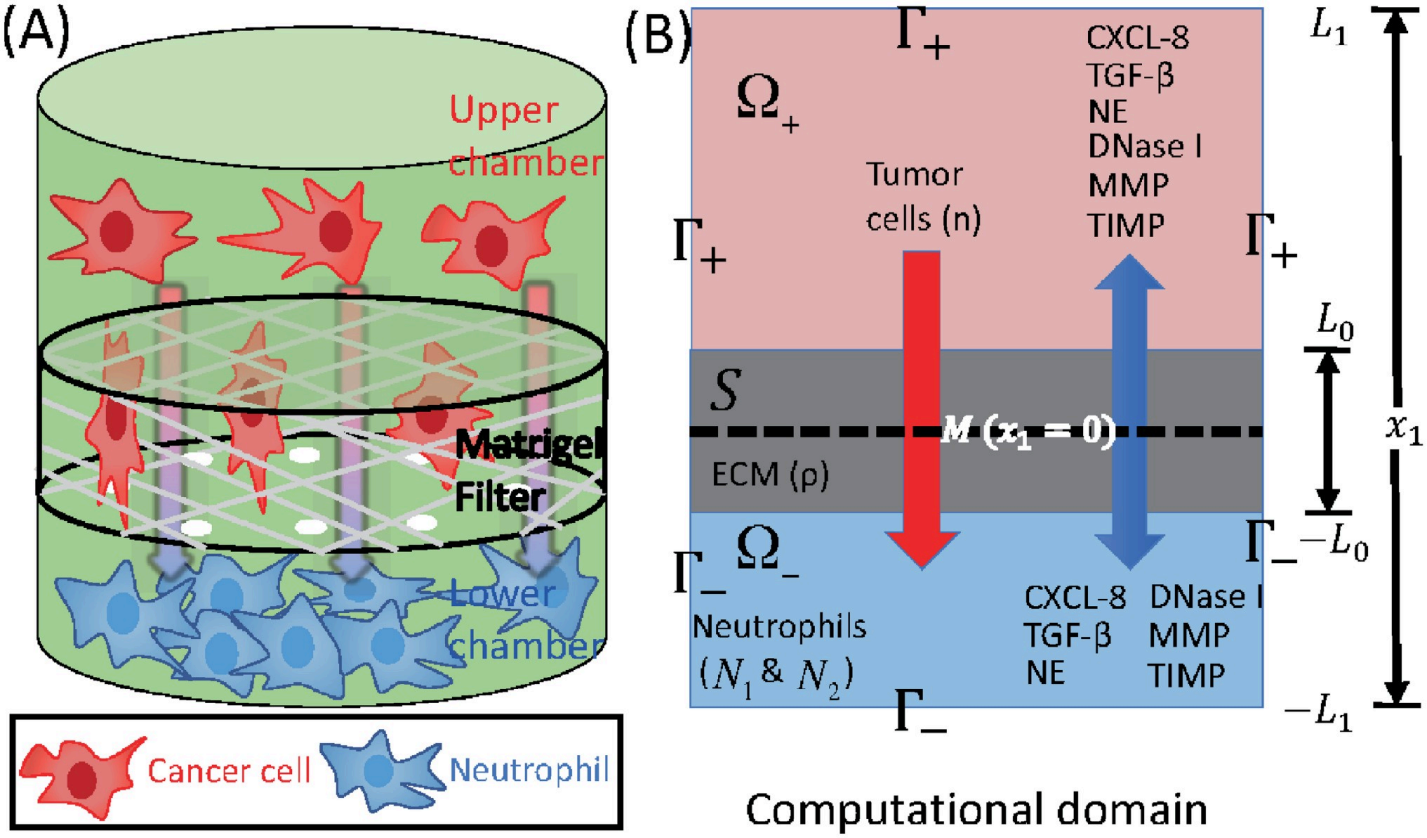
Schematics of an invasion assay system. (A) Boyden transwell invasion assay. Tumor cells were suspended in the upper chamber, while neutrophils or medium alone (control) were placed in the lower chamber. Semipermeable inserts coated with matrigel (extracellular matrix) were inserted in the filter. In response to NE secreted by N2 neutrophils in the lower chamber, tumor cells degrade the heavy extracellular matrix proteolytically and invade the lower chamber. The number of neutrophils on the lower surface of the permeable insert was counted after 22*h* in the absence and presence of neutrophils in the lower chamber. (B) TGF-*β* (*G*), NE (*E*), NE inhibitor (*D*), CXCL8 (*C*), MMP (*P*), TIMP (*M*) and tumor cells (*n*) can cross the semi-permeable membrane, but neither type of neutrophils (*N*_1_, *N*_2_) can cross it. Initially, the tumor cells reside in the upper chamber (domain Ω_+_) while neutrophils are placed in the lower chamber (domain Ω_−_). An extracellular matrix (ECM) layer (*S*) surrounds the filter, semi-permeable membrane (M).

Mathematical models of tumor microenvironment and tumor-immune system interactions have been developed: fibroblasts-tumor [33–35], macrophages-tumor [36, 37], astrocytes-tumor [38], NK cells-tumor [39–41], neutrophil-tumor [42, 43], tumor-endothelial [44], and immune-tumor [45, 46] interactions. However, the detailed mechanism of tumor invasion and metastasis via communication with TANs is still poorly understood. It would be difficult to build a comprehensive mathematical model of the tumor invasion and metastasis that incorporates all the biochemical and mechanical processes (S1 Text). As a beginning step, we focus on the neutrophil-mediated invasion of tumor cells, for which there are experimental data. Here, we develop a mathematical model based on taxis-reaction-diffusion equations that govern cell-cell signaling and chemotactic cell movement. Our goal is to understand the biochemical factors that are important in regulating the chemotactic movement of tumor cells from the upper chamber to the lower well of the Boyden chamber assay shown in Fig 2. We show that the mathematical model can replicate the major components of experimental findings and we test several anti-invasion intervention strategies with predictions.

## Materials and methods

We developed a mathematical model of tumor cell invasion in *in vitro* experiments, a critical step in metastasis [22, 47, 48], based on mutual interactions between tumor cells and neutrophils (Fig 1).

We denote by Ω the 3-dimensional domain

$$\begin{array}{c} \Omega =\{x=\left(x_{1},x_{2},x_{3}\right);\;-L_{i}<x_{i}<L_{i}\;\text{for}\;1\leq i\leq 3\} \end{array}$$

and set

$$\begin{array}{ccc} & & \Omega_{+}=\Omega \cap \left\{x_{1}>0\right\},\;\;\Omega_{-}=\Omega \cap \left\{x_{1}<0\right\},\;\;\Omega_{*}=\Omega_{+}\cup \Omega_{-},\\ & & \Gamma_{+}=\partial \Omega_{+},\;\;\Gamma_{-}=\partial \Omega_{-}. \end{array}$$

The semi-permeable membrane occupies the planar region

$$\begin{array}{c} M=\{x_{1}=0,\;-L_{i}<x_{i}<L_{i}\;\text{for}\;i=2,3\}, \end{array}$$

and the ECM occupies a 3-dimensional region

$$\begin{array}{c} S=\{-L_{0}<x_{1}<L_{0},\;x_{1}\neq 0,\;-L_{i}<x_{i}<L_{i}\;\text{for}\;i=2,3\} \end{array}$$

where 0 < *L*_0_ < *L*_1_. We denote by *I*_*A*_ the characteristic function of a set *A*:

$$\begin{array}{c} I_{A}\left(x\right)=1\;\text{if}\;x\in A,\;\;\;\;I_{A}\left(x\right)=0\;\text{if}\;x\notin A \end{array}$$

The geometry of the experimental setup of the Boyden invasion chamber is shown in Fig 2A. In the typical transwell migration assay, neutrophils isolated from the bone marrow are plated in the lower chamber, and tumor cells are added on top of Matrigel-coated insert in the upper chamber [22]. In our model, we assume that tumor cells are initially placed on the top of gel-coated area above the membrane with mini-pores in the middle, and invade the lower chamber where neutrophils (or conventional medium for control) reside. The corresponding computational domain is shown in Fig 2B.

We introduce the following variables at space **x** and time *t*:

*n*(**x**, *t*) = density of tumor cells,
*N*_1_(**x**, *t*) = density of N1 neutrophils,
*N*_2_(**x**, *t*) = density of N2 neutrophils,
*ρ*(**x**, *t*) = concentration of tumor extracellular matrix (ECM),
*C*(**x**, *t*) = concentration of CXCL-8,
*G*(**x**, *t*) = concentration of TGF-*β*,
*E*(**x**, *t*) = concentration of NET/NE,
*D*(**x**, *t*) = concentration of NET/NE inhibitor,
*P*(**x**, *t*) = concentration of MMPs,
*M*(**x**, *t*) = concentration of MMP inhibitor (TIMP).

The evolution equations for these variables are developed in next sections, but in this work we focus on the Boyden invasion chamber, transwell assay, in one space dimension.

### Tumor cell density (= *n*(x, *t*))

The mass balance equation for the tumor cell density *n*(**x**, *t*) is

$$\begin{array}{c} \frac{\partial n}{\partial t}=-\nabla \cdot J_{n}+P_{n}, \end{array}$$ (1)

where **J**_*n*_ is the flux and *P*_*n*_ is the net production rate of cancer cells. The flux **J**_*n*_ is comprised of three components, **J**_*random*_, **J**_*chemo*_, and **J**_*hapto*_, which are the fluxes due to random motion, chemotaxis, and haptotaxis, respectively [34, 49].

We assume that the tumor extracellular matrix is homogeneous and isotropic in tumor microenvironment, and that the flux due to the random motility is given by

$$\begin{array}{c} J_{random}=-D_{n}\nabla n \end{array}$$ (2)

where *D*_*n*_ is the random motility constant of tumor cells.

In lung tissue, tumor cells are strongly attracted to chemotactic attractants [32] such as NE and neutrophils [22, 32] and migrate toward the up-gradient (∇*E*) of the chemo-attractant, NE, through the process called *‘chemotaxis’* [50]. The chemotactic flux is assumed to be of the form

$$\begin{array}{c} J_{chemo}=\chi_{E}\;n\;\frac{\nabla E}{\delta_{E}+\sigma_{E}\left|\nabla E\right|}, \end{array}$$ (3)

where *χ*_*E*_ is the chemotactic sensitivity, *δ*_*E*_, *σ*_*E*_ are scaling parameters, and *E* is the concentration of NE, whose dynamics will be introduced in Section below. This form reduces to the standard form of the chemotactic flux (**J**_*chemo*_ ≈ *C n* ∇*E*; *C* = constant) under small NE gradients (|∇*E*| ≪ 1) and saturates (**J**_*chemo*_ ≈ (*χ*_*E*_/*δ*_*E*_) *n* **u**; **u** = ∇*E*/|∇*E*| is the unit vector) under large NE gradients, preventing the blow-up behaviors of solutions [34]. Other forms such as $\chi_{n}\frac{\nabla E}{E}$ [51] or $\chi_{n}\frac{C}{{\left(C+E\right)}^{2}}\nabla E$ (*C*: constant) [52] have been adapted in the literature.

Tumor cell invasiveness is enhanced by proteolytic degradation of the extracellular matrix via MMPs [4, 25, 53] and NEs [29, 54] that are produced by neutrophils. This results in local degradation of tumor ECM [49] and tumor cell movement in the direction of the up-gradient (∇*ρ*) of ECM via a cellular process called *haptotaxis*. This process is valid only in the ECM domain *S*, therefore, we include the characteristic function *I*_*S*_, providing the on-off switch on the ECM membrane. We represent the haptotactic flux in a similar fashion:

$$\begin{array}{c} J_{hapto}=\chi_{\rho }I_{S}\;n\;\frac{\nabla \rho }{\delta_{\rho }+\sigma_{\rho }\left|\nabla \rho \right|}, \end{array}$$ (4)

where *χ*_*ρ*_ is the haptotactic sensitivity, *δ*_*ρ*_, *σ*_*ρ*_ are scaling parameters, and *ρ* is the concentration of tumor ECM, whose dynamics will be introduced in Section below.

The net production of tumor cells is due to active NE-stimulated growth [8, 22] and cell killing by N1 TANs [3, 21, 23], which we represent as follows:

$$\begin{array}{c} P_{n}=r(1+r_{E}\frac{E^{m}}{k_{E}^{m}+E^{m}})n(1-\frac{n}{n_{0}})-\mu_{n}N_{1}n. \end{array}$$ (5)

Here *r* is the proliferation rate of tumor cells in the absence of NE (*E*), *r*_*E*_ is the dimensionless parameter of NE-mediated tumor growth, *k*_*E*_ and *m* are Hill-function coefficients for activation of proliferation in the presence of NE, *n*_0_ is the carrying capacity of the tumor in a given TME, and, finally *μ*_*n*_ is the killing rate of tumor cells by N1 neutrophils (*N*_1_) whose dynamics will be described in Section *‘Densities of neutrophils’* below. Here, $r,r_{E},k_{E},n_{0},\mu_{n}\in R^{+}$, $m\in Z^{+}$.

Combining the several fluxes in Eqs (2)–(4) and growth term in Eq (5) leads to the governing equation for the tumor cell density

$$\begin{array}{ccc} & & \frac{\partial n}{\partial t}=\nabla \cdot (D_{n}\nabla n-\chi_{E}n\frac{\nabla E}{\delta_{E}+\sigma_{E}\left|\nabla E\right|}-\chi_{\rho }I_{S}n\frac{\nabla \rho }{\delta_{\rho }+\sigma_{\rho }\left|\nabla \rho \right|})\\ & & \;\;+r(1+r_{E}\frac{E^{m}}{k_{E}^{m}+E^{m}})n(1-\frac{n}{n_{0}})-\mu_{n}N_{1}n\;\;\;\text{in}\;\Omega_{*},\;t>0. \end{array}$$ (6)

### Densities of neutrophils: N1 (= *N*_1_(x, *t*)) & N2 (= *N*_2_(x, *t*)) types

We use a similar form of reaction-diffusion-advection equations for the evolution of the densities of neutrophils, based on mass balance as in the previous section. We assume that (i) Neutrophils are chemotactic to the CXCL secreted by tumor cells [55–57], and the chemotactic flux is of the nonlinear form (3), but with different chemotactic sensitivities (*χ*_1_, *χ*_2_). Since the N2 TANs produce NE and MMPs, the movement of activated neutrophils further enhances tumor invasiveness and growth via the NE-PI3K pathway described earlier. (ii) The anti-tumorigenic (N1) neutrophils transform into the active N2 type at the rate λ_12_ in the presence of TGF-*β*, based on experimental evidences [3, 21, 43]. For instance, the N1→N2 transition of TANs with protumour properties was typically observed in a TGF-*β*-rich tumor microenvironment and the presence of IFN-*β* or TGF-*β* inhibitor can mediate the reverse transition (N2→N1) with anti-tumoral properties [3]. Therefore, TGF-*β* pathway inhibitors are under clinical trials since they were shown to promote the development of N1 TANs [58, 59]. (iii) N1 and N2 phenotypes proliferate at a rate, λ_1_ and λ_2_(*G*), respectively. Then, we have the following evolution equations:

$$\begin{array}{ccc} & & \frac{\partial N_{1}}{\partial t}=\nabla \cdot (D_{1}\nabla N_{1}-\chi_{1}N_{1}\frac{\nabla C}{\delta_{1}+\sigma_{C}\left|\nabla C\right|})+\lambda_{1}N_{1}-\lambda_{12}GN_{1}\;\;\text{in}\;\Omega_{*},\;t>0, \end{array}$$ (7)

$$\begin{array}{ccc} & & \frac{\partial N_{2}}{\partial t}=\nabla \cdot (D_{2}\nabla N_{2}-\chi_{2}N_{2}\frac{\nabla C}{\delta_{2}+\sigma_{C}\left|\nabla C\right|})+\lambda_{12}GN_{1}+\lambda_{2}\left(G\right)N_{2}\;\;\;\;\;\text{in}\;\Omega_{*},\;t>0. \end{array}$$ (8)

### Tumor ECM density (= *ρ*(x, *t*))

The tumor ECM provides structural foundation for efficient cell migration [60], but it also needs to be remodeled via proteolysis for tumor cell migration by microenvironmental proteases [55, 61–63]. In this work, we assume that the tumor ECM is degraded by the TAN-secreted NEs [64] and TAN-secreted MMPs [4, 25, 53, 55] as in the invasion experiments [22]. The rate of ECM change can be represented as

$$\begin{array}{c} \frac{d\rho }{dt}=-\left(\mu_{\rho 1}E+\mu_{\rho 2}P\right)n\;\;\;\text{in}\;S,\;t>0. \end{array}$$ (9)

Here *μ*_*ρ*1_, *μ*_*ρ*2_ are the degradation rates by NEs and MMPs, respectively, which are secreted by N2 neutrophils. Essentially, this equation represents proteolytic degradation of tumor ECM coated on the filter when there is a significant level of tumor ECM present, as is normally the case in a TME.

### CXCL8 concentration (= *C*(x, *t*))

Tumor cells secrete CXCL in order to recruit the immune cells such as neutrophils [55–57]. CXCLs and corresponding receptors (CXCR) such as CXCL5 and CXCR6 are important prognostic factors, alone or in a combination with the TANs, for shorter overall survival and cumulative risk of recurrence [3, 65, 66]. Thus the governing equation for CXCL8 is

$$\begin{array}{ccc} & & \frac{\partial C}{\partial t}=D_{C}\Delta C+\lambda_{C}n-\mu_{C}C\;\;\;\text{in}\;\Omega_{*},\;t>0, \end{array}$$ (10)

where *D*_*C*_ is the diffusion coefficient of CXCL, λ_*C*_ is the secretion rate of CXCL by tumor cells and *μ*_*C*_ is the decay rate of CXCL.

### TGF-*β* concentration (= *G*(x, *t*))

TGF-*β* is a polypeptide that plays a major role in regulation of many human diseases including cancers [67] due to its capacity of maintaining tissue homeostasis and involving in most of the chronic inflammatory and wounding processes by activating its inactive form in ECM [68]. Tumor cells are the primary source of TGF-*β* in TME [3, 69]. TGF-*β* activates proinflammatory and antitumorigenic N1 neutrophils into the aggressive N2 type, which in turn stimulates tumor cell invasion [55, 70]. Thus the governing equation for TGF-*β* is as follows:

$$\begin{array}{c} \frac{\partial G}{\partial t}=D_{G}\Delta G+\lambda_{G}n-\mu_{G}G\;\;\;\text{in}\;\Omega_{*},\;t>0, \end{array}$$ (11)

where *D*_*G*_ is the diffusion coefficient of TGF-*β*, λ_*G*_ is the secretion rate of TGF-*β* by tumor cells, and *μ*_*G*_ is the decay rate of TGF-*β*.

### Concentrations of NET/NE (= *E*(x, *t*)) and its inhibitors (= *D*(x, *t*))

NET and NE are highly associated with aggressive invasion, growth, EMT, and metastasis of cancer cells [4, 54, 71]. NE is produced by neutrophils [4, 64, 72] and used for degradation of extracellular matrix and tissue destruction [8, 54, 73]. It was also shown that NE inhibitors such as DNase I block this effect in growth models [8] and invasion assays [22]. In our framework, NET and the associated NEs are merged into one component. Thus, the governing equations for NET/NE and its inhibitors are

$$\begin{array}{ccc} & & \frac{\partial E}{\partial t}=D_{E}\Delta E+\lambda_{E}N_{2}-\mu_{E}E-\mu_{ED}\frac{ED^{l}}{K_{D}^{l}+D^{l}}\;\;\;\text{in}\;\Omega_{*},\;t>0, \end{array}$$ (12)

$$\begin{array}{ccc} & & \frac{\partial D}{\partial t}=D_{D}\Delta D+\lambda_{D}I_{\Omega_{I}}-\mu_{D}D\;\;\;\;\;\;\;\;\;\text{in}\;\Omega_{*},\;t>0, \end{array}$$ (13)

where *D*_*E*_, *D*_*D*_ are diffusion coefficients of NET/NE and its inhibitors, respectively, λ_*E*_ is the production rate of NET/NE from N2 neutrophils, λ_*D*_ is the injection rate of NET/NE inhibitors at a subdomain Ω_*I*_, *μ*_*E*_, *μ*_*D*_ are natural decay rates of NET/NE and its inhibitors, respectively, *μ*_*ED*_ is the consumption rate of NE in response to NE inhibitors with kinetic parameters *K*_*D*_, *l* ($D_{E},D_{D},\lambda_{E},\lambda_{D},\mu_{E},\mu_{D},\mu_{ED},K_{D}\in R^{+}$, $l\in Z^{+}$).

### MMP concentration (= *P*(x, *t*))

Matrix metalloproteinases (MMPs) are highly associated with cancer cell invasion and metastasis [48, 74]. Neutrophils, not tumor cells [55, 75], were suggested to the primary source of MMPs [4, 25, 53, 55] including MMP-9 [53] in lung cancer development, showing strikingly predominant presence at the invasive fronts of metastatic cancers [53]. Thus the governing equation for MMPs is

$$\begin{array}{c} \frac{\partial P}{\partial t}=D_{P}\Delta P+\lambda_{P}N_{2}-\mu_{PM}\frac{PM^{m}}{K_{M}^{m}+M^{m}}-\mu_{P}P\;\;\;\text{in}\;\Omega_{*},\;t>0, \end{array}$$ (14)

where *D*_*P*_ is the diffusion coefficient of MMPs, λ_*P*_ is the MMP production rate by N2 neutrophils, *μ*_*PM*_ is the degradation of MMPs by its inhibitor, TIMP, with Hill-coefficients *K*_*M*_, *m* ($K_{M}\in R^{+},m\in Z^{+}$), *μ*_*P*_ is the decay rate of MMPs. In general, *D*_*P*_ is very small (*D*_*P*_ ≪ 1) while the half-life of MMPs is short (*μ*_*P*_ ≫ 1) [76], leading localized activities at the moving front of invasive cells.

### TIMP concentration (= *M*(x, *t*))

Tissue inhibitors of metalloproteinases (TIMPs) play an important role in inhibiting tumor invasion and metastasis [77] by regulating major signaling pathways in pericellular proteolysis of various extracellular matrix and cell surface proteins [78]. In the model, TIMPs are injected for inhibition of the proteolytic activities of cancer cell invasion. Note, however, that this action can partially block cancer cell invasion since cancer cells can still execute the NE-mediated invasion. Thus, the governing equation of TIMP is

$$\begin{array}{c} \frac{\partial M}{\partial t}=D_{M}\Delta M+\lambda_{M}-\mu_{M}M\;\;\;\text{in}\;\Omega_{*},\;t>0, \end{array}$$ (15)

where *D*_*M*_ is the diffusion coefficient, λ_*M*_ is the TIMP supply rate, and *μ*_*M*_ is the decay rate of TIMP.

### Boundary conditions and initial conditions

In the following simulations we prescribe Neumann boundary conditions on the exterior boundary Γ_1_ (= ∂Ω; see Fig 2B) as follows:

$$\begin{array}{cc} & J_{n}\cdot \nu =0,\\ & (D_{1}\nabla N_{1}-\chi_{1}\;N_{1}\;\frac{\nabla C}{\delta_{1}+\sigma_{C}\left|\nabla C\right|})\cdot \nu =0,\\ & (D_{2}\nabla N_{2}-\chi_{2}\;N_{2}\;\frac{\nabla C}{\delta_{2}+\sigma_{C}\left|\nabla C\right|})\cdot \nu =0,\\ & (D_{C}\nabla C)\cdot \nu =0,\;\;\;(D_{G}\nabla G)\cdot \nu =0,\\ & (D_{E}\nabla E)\cdot \nu =0,\;\;\;(D_{P}\nabla P)\cdot \nu =0,\\ & (D_{D}\nabla D)\cdot \nu =0,\;\;\;(D_{M}\nabla M)\cdot \nu =0, \end{array}$$ (16)

where *ν* is the unit outer normal vector. The membrane is permeable to all variables (*n*, *N*_1_, *N*_2_, *C*, *G*, *E*, *D*, *P*, *M*), but not freely so. We describe the flux at the membrane boundary Γ_2_ (= M; see Fig 2B) for these variables **u** = (*n*, *N*_1_, *N*_2_, *C*, *G*, *E*, *D*, *P*, *M*) as

$$\begin{array}{ccc} & & J^{+}=J^{-},\;\;J^{+}+\gamma_{i}\left(u^{+}-u^{-}\right)=0, \end{array}$$ (17)

where

$$\begin{array}{c} u\left(x\right)=\{\begin{array}{cc} u^{+}\left(x\right) & \;\;\;\text{if}\;x_{1}>0\\ u^{-}\left(x\right) & \;\;\;\text{if}\;x_{1}<0. \end{array} \end{array}$$ (18)

Here, the parameters *γ*_*i*_ (*γ*_*i*_ > 0, *i* = 1, ⋯, 9) represent the permeability of cells (*i* = 1, 2, 3) and molecules (*i* = 4, ⋯, 9). The permeability (*γ*_*i*_) is determined by the density and size of the holes on the semi-permeable membrane between upper and lower chambers as well as the size of the moving object relative to the hole size. The holes in the insert are uniformly distributed on the membrane of the Boyden invasion transwell assay [22, 31]. See [79] for the derivation of these Robin-type boundary conditions by the homogenization method. If the size of the circular holes in the membrane is increased (or decreased), the membrane becomes more (or less) permeable, and *γ*_*i*_ increases (or decreases) [33, 34]. For instance, the diameter of typical cells is in the range of 10-20 *μm* while the size of growth factors and cytokines such as TGF-*β* is much smaller [80]. Furthermore, the diffusion coefficient of cells is usually much smaller than that of growth factors and cytokines [81]. While, the typical diffusion coefficient of molecules (CXCL8, EGF, and TGF-*β*) is in the range of (1.0-2.5) × 10^−6^ *cm*^2^/*s* [57, 82–86], the random motility coefficient of cells is much smaller ((1.0-10.0) × 10^−10^ *cm*^2^/*s*). Therefore, the parameter (*γ*_*i*_ = *γ*_*c*_ (*i* = 1, 2, 3)) of the migratory cells is smaller than the permeability parameter (*γ*_*i*_ = *γ* (*i* = 4, ⋯, 9)) of the diffusible molecules due to different physical sizes. In a classical Boyden invasion chamber, a typical, invasive tumor cell in the upper chamber is not able to invade the lower chamber if the diameter of the permeable holes on the membrane is less than 0.4 *μm* while molecules can diffuse throughout the domain [33]. So, we take the smaller permeability parameter for cells (*γ*_*c*_ < *γ*).

Finally, we prescribe initial conditions,

$$\begin{array}{ccc} & & n\left(x,0\right)=n_{0}\left(x\right)\;\;\;\text{in}\;\;\Omega_{*},\\ & & N_{1}\left(x,0\right)=N_{10}\left(x\right),\;N_{2}\left(x,0\right)=N_{20}\left(x\right)\;\;\;\text{in}\;\;\Omega_{*},\\ & & \rho \left(x,0\right)=\rho_{0}\left(x\right)\;\;\;\text{in}\;\;S,\\ & & \;C\left(x,0\right)=C_{0}\left(x\right),\;G\left(x,0\right)=G_{0}\left(x\right),\;E\left(x,0\right)=E_{0}\left(x\right),\;D\left(x,0\right)=D_{0}\left(x\right),\;\;\;\text{in}\;\Omega_{*},\\ & & \;P\left(x,0\right)=P_{0}\left(x\right),\;M\left(x,0\right)=M_{0}\left(x\right)\;\;\;\text{in}\;\Omega_{*}. \end{array}$$ (19)

Parameters are given in Tables 1 and 2. Nondimensionalization and parameter estimation of the system (6)–(19) are given in S2 and S3 Text, respectively. This non-dimensional form of governing equations was used for the simulations. Hereafter, the computational domain is restricted to one space dimension, and the computational domain is scaled to unit length.

**Table 1:** Parameters used in the tumor model.

	Description	Dimensional Value	Refs.
Diffusion coefficients (*cm*^2^ *s*^−1^)			
*D*_*n*_	Tumor cells	2.5 × 10^−8^	[33, 37, 129–131]
*D*_1_	N1 Neutrophil	1.1 × 10^−8^	[83]
*D*_2_	N2 Neutrophil	1.1 × 10^−8^	[83]
*D*_*C*_	CXCL8 (IL-8) chemokines	2.5 × 10^−6^	[57, 82, 83]
*D*_*G*_	TGF-*β*	1.0 × 10^−6^	[84–86]
*D*_*E*_	NET/NE	5.0 × 10^−7^	[132–137]
*D*_*P*_	MMP	5.0 × 10^−10^	[37, 138, 139]
*D*_*D*_	DNase I (NET/NE inhibitor)	7.374 × 10^−6^	[140], estimated
*D*_*M*_	TIMP (MMP inhibitor)	8.33 × 10^−7^	estimated
*D*_*A*_	TGF-*β* Anti-body	8.33 × 10^−7^	estimated
Production rates			
*r*	Proliferation rate of tumor cells	3.3 × 10^−4^ *s*^−1^	[22, 33], estimated
*r*_*E*_	NE-mediated proliferation rate of tumor cells	7.0 × 10^−2^	estimated
*k*_*E*_	Hill type coefficient of tumor cell proliferation	2.15 × 10^−9^ *gcm*^−3^	[33], estimated
*m*	Hill coefficient of NE-mediated tumor proliferation	2	estimated
*n*_0_	Tumor cell carrying capacity	2.5 × 10^4^ *cells*/*cm*^3^	[33], estimated
λ_1_	Proliferation rate of N1 neutrophil	4.38 × 10^−6^ *s*^−1^	[33, 141], estimated
λ_12_	Transformation rate from N1 to N2 neutrophils	4.08 × 10^3^ *cm*^3^ *g*^−1^ *s*^−1^	[33], estimated
λ_2_	Proliferation rate of N2 neutrophils	2.65 × 10^−5^ *s*^−1^	[33, 141], estimated
λ_*C*_	Production rate of CXC from tumor cells	4.44 × 10^−11^ *s*^−1^	[33, 142, 143], estimated
λ_*G*_	Production rate of TGF-*β* from tumor cells	4.89 × 10^−7^ *s*^−1^	[33, 142, 143]
λ_*E*_	Production rate of NET/NEs from Neutrophils	2.26 × 10^−7^ *s*^−1^	[144]
λ_*P*_	Production rate of MMPs from Neutrophils	2.22 × 10^−8^ *s*^−1^	[144]
λ_*D*_	Production rate of NET/NE inhibitor	9.0 × 10^−13^ *gcm*^−3^ *s*^−1^	estimated
λ_*M*_	Production rate of TIMP	1.29 × 10^−11^ *gcm*^−3^ *s*^−1^	estimated
λ_*A*_	Production rate of TGF-*β* anti-body	4.78 × 10^−5^ *μMs*^−1^	estimated
Degradation/Decay rates			
*μ*_*n*_	tumor cells degradation rate by N1	2.78 × 10^−1^ *cm*^3^ *g*^−1^ *s*^−1^	estimated
*μ*_*ρ*1_	ECM degradation rate by NEs	1.02 × 10^4^ *cm*^3^ *g*^−1^ *s*^−1^	estimated
*μ*_*ρ*2_	ECM degradation rate by MMPs	3.19 × 10^5^ *cm*^3^ *g*^−1^ *s*^−1^	estimated
*μ*_*C*_	decay rate of CXCL8	6.42 × 10^−5^ *s*^−1^	[145–147]
*μ*_*G*_	decay rate of TGF-*β*	8.02 × 10^−6^ *s*^−1^	[33], estimated
*μ*_*E*_	decay rate of neutrophil elastase	8.02 × 10^−6^ *s*^−1^	[148]
*μ*_*P*_	decay rate of MMP	5.0 × 10^−5^ *s*^−1^	[49]
*μ*_*D*_	decay rate of NE inhibitor	9.627 × 10^−5^ *s*^−1^	[149–153]
*μ*_*M*_	decay rate of TIMP	4.56 × 10^−6^ *s*^−1^	[154]
*μ*_*ED*_	degradation rate of NET/NE by its inhibitor	(2.8 × 10^−4^—2.8 × 10^−2^) *s*^−1^	estimated
*μ*_*A*_	decay rate of TGF-*β* anti-body	6.42 × 10^−5^ *s*^−1^	[43, 155]
*μ*_*AG*_	decay rate of TGF-*β* by anti-body	4.8 × 10^−3^ *μM*^−1^ *s*^−1^	[43], estimated
*K*_*D*_	Hill coefficient of NET/NE degradation by its inhibitor	3.2 × 10^−9^ *g*/*cm*^3^	[156], estimated
*l*	Hill coefficient of NET/NE degradation by its inhibitor	2	[156], estimated
*μ*_*PM*_	degradation rate of MMPs by TIMP	(2.8 × 10^−5^—2.8 × 10^−4^) *s*^−1^	estimated

**Table 2:** Parameters used in the tumor model. **Continued from** Table 1.: Dimensionless values were marked in *.

	Description	Dimensional Value	Refs.
Degradation/Decay rates			
*K*_*M*_	Hill coefficient of MMP degradation by TIMP	4.64 × 10^−8^ *g*/*cm*^3^	[156], estimated
*m*	Hill coefficient of MMP degradation by TIMP	2	estimated
Movement parameters (chemotaxis, haptotaxis)			
*χ*_*E*_	Chemotactic sensitivity to NE	1.117 × 10^−9^ *cm*.*s*^−1^	[22, 33, 130, 157]
*δ*_*E*_	Scaling parameter of NE gradient (|*E*|)	6.46 × 10^−8^ *g*/*cm*^4^	estimated
*σ*_*E*_	Scaling parameter of NE gradient (|*E*|)	1.0	estimated
*χ*_*ρ*_	Haptotactic sensitivity	3.5 × 10^−10^ *cm*.*s*^−1^	[22, 158–160], estimated
*δ*_*ρ*_	Scaling parameter of ECM gradient (|*ρ*|)	5.0 × 10^−3^ *g*/*cm*^4^	estimated
*σ*_*ρ*_	Scaling parameter of ECM gradient (|*ρ*|)	1.0	estimated
$\chi_{1}^{C}$	Chemotactic sensitivity of N1 neutrophils to CXCL8	1.1 × 10^−9^ *cm*.*s*^−1^	[22, 33, 161], estimated
*δ*_1_	Scaling parameter of CXCL gradient (|*C*|)	1.0 × 10^−11^ *g*/*cm*^4^	estimated
$\chi_{2}^{C}$	Chemotactic sensitivity of N2 neutrophils to CXCL8	1.1 × 10^−9^ *cm*.*s*^−1^	[22, 33, 161], estimated
*δ*_2_	Scaling parameter of CXCL gradient (|*C*|)	1.0 × 10^−11^ *g*/*cm*^4^	estimated
*σ*_*C*_	Scaling parameter of CXCL8 gradient (|*C*|)	1.0*	estimated
Membrane parameters			
*γ*_*c*_	permeability of cells (Tumor cells, N1 neutrophils, N2 neutrophils)	0.01-785 (78.5)*	estimated
*γ*	permeability of chemicals (CXCL8, TGF-*β*, NET/NE, NE inhibitor, MMP, TIMP)	0.1-7850 (785)*	[33], estimated
*μ*	ECM width	0.6*	estimated

All the simulations were performed using a finite volume method (FVM; clawpack (http://www.amath.washington.edu/~claw/)) with fractional step method [87]. A nonlinear solver *nksol* was used for solving algebraic systems. The Eqs (6)–(19) were solved on a regular uniform grid with grid size 0.01 (*h*_*x*_ = 0.01). An initial time step of 0.0001 (or smaller) was used, but adaptive time stepping scheme based on the number of iterations can increase or decrease this step size.

## Results

In this section, we investigate the role of NEs in regulation of cancer cell invasion, compare the predictions of our mathematical model with experimental data, and then suggest new therapeutic strategies for blocking invasive tumor cells.

### Predictions of the mathematical model

**Fig 3:**
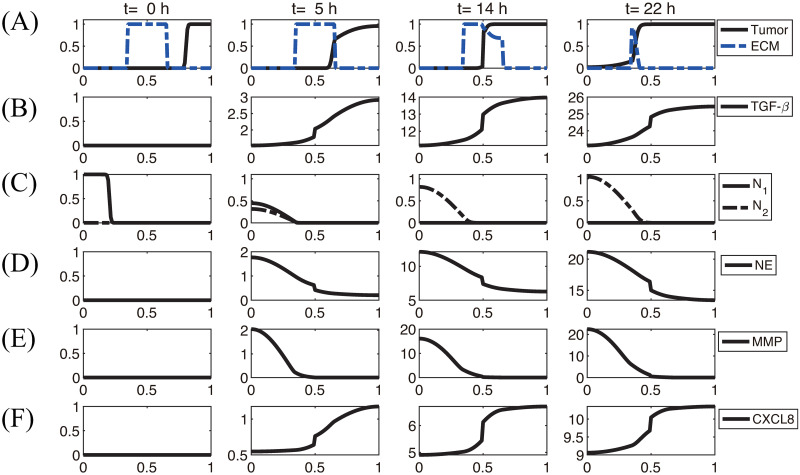
Dynamics of the system. The time evolution of the density of each variable. (A) tumor cells and ECM (B) TGF-*β* (C) N1/N2 neutrophils (D) neutrophil elastase (E) MMP (F) CXCL8. Here, ECM = [0.35, 0.65]⊂ Ω = [0, 1]. Note that the initial concentrations of CXCL8, TGF-*β*, neutrophil elastase and MMPs are uniformly zero, as in experiments. *x-axis = space (the dimensionless length across the invasion chamber), y-axis = the dimensionless density/concentration of the variables.

Fig 3 shows the density profiles of all variables (*n*, *N*_1_, *N*_2_, *ρ*, *C*, *G*, *E*, *P*) at *t* = 0, 5, 14, 22*h* in the absence of DNase I and TIMP when neutrophils were added in the lower chamber. In each subframe, the right (or left) half of the computational domain represents the upper (or lower) chamber in the Boyden invasion assay (Fig 2A). By degradation of the tumor ECM on the membrane of the insert, tumor cells in the upper chamber were experimentally shown to have capacity of invading the lower chamber upon stimulus of N1/N2 neutrophils in the lower chamber [22, 31]. Tumor cells in the upper chamber secrete CXCL8 (Fig 3F), which then diffuses and attracts neutrophils in the lower chamber by *chemotaxis*. On the other hand, tumor cells produce TGF-*β* (Fig 3B), which diffuses and enhances the N1 → N2 transformation of neutrophils (Fig 3C) in the lower chamber. These activated N2 TANs in the lower chamber then secrete NET/NEs (Fig 3D) and MMPs (Fig 3E) to stimulate chemotactic and haptotactic movement of tumor cells in the upper chamber. Tumor cells break down the ECM component by proteolytic activities with the NE and MMP near the membrane and invade the left chamber (Fig 3A). As they invade, they can sense higher levels of NE, and proliferate at a higher rate (see Eq (6)).

**Fig 4:**
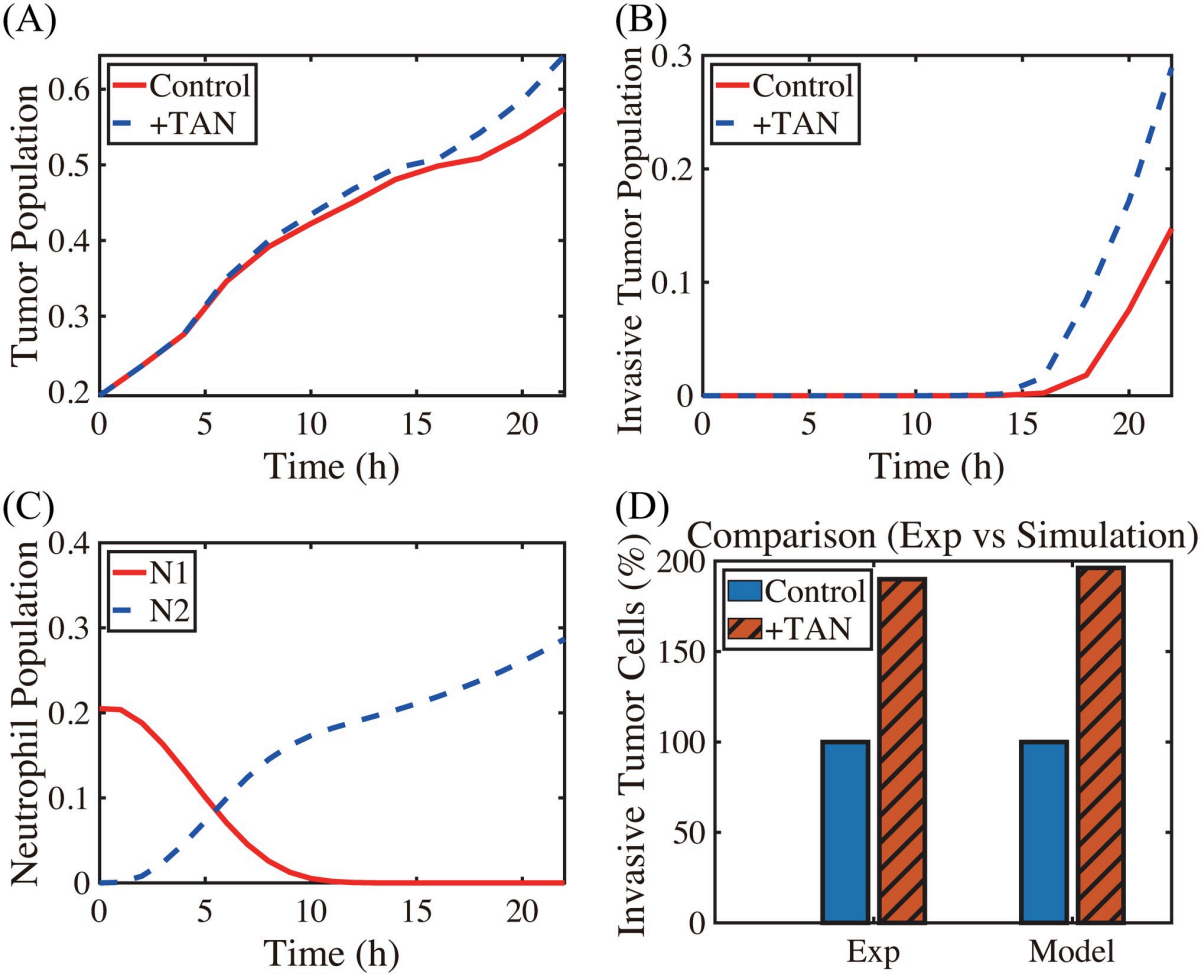
TAN-promoted cancer cell invasion (Experiment [22] & simulation). (A-B) Time courses of populations of total tumor cells (A) and invasive tumor cells (B). (C) Time courses of N1 (red solid) and N2 (blue dashed) neutrophils. (D) Experimental data from the invasion assay in [22] (left column; 4T1 cancer cells) and computational results from mathematical model (right column). The graph shows the (scaled) populations of invasive tumor cells at *t* = 22 *h* in the absence (control) or presence (^+^TAN) of neutrophils. Here and hereafter cell populations are derived from the continuum density.

**Fig 5:**
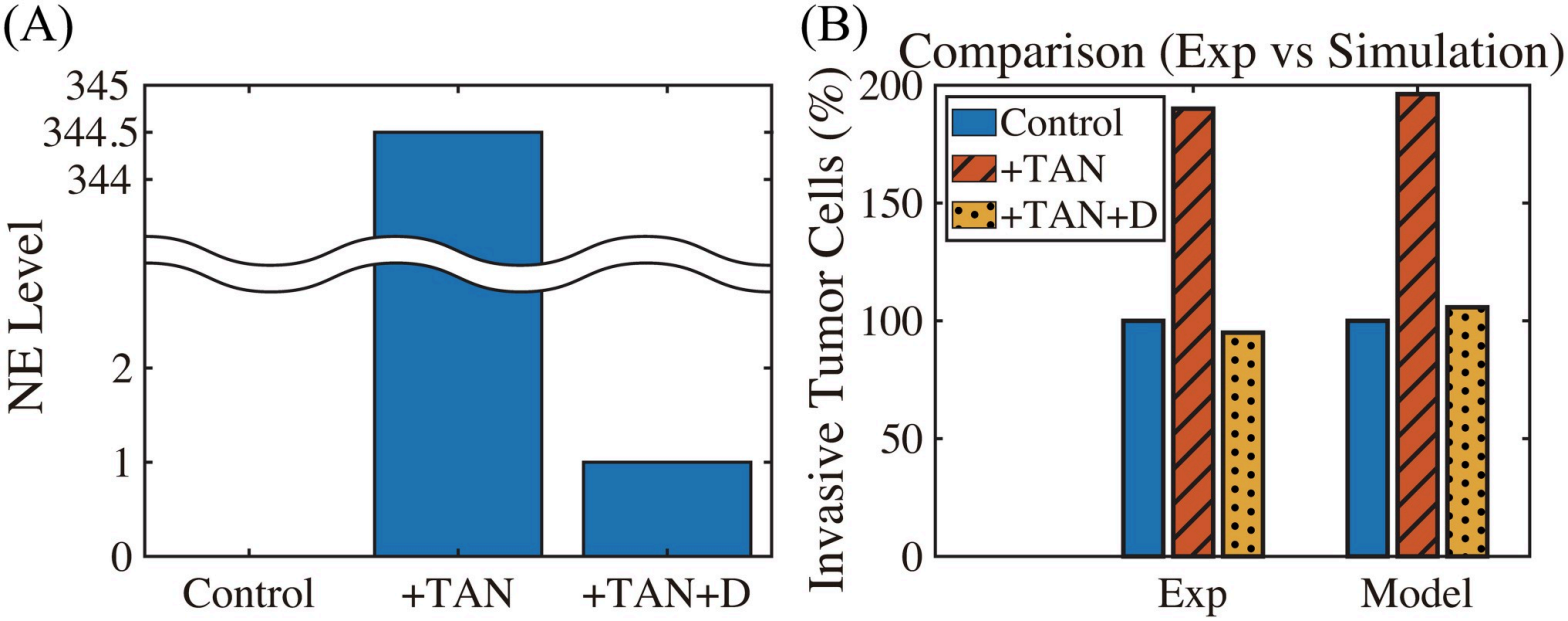
DNase I treatment against NE can abrogate the invasion-boosting effects of neutrophils (Experimental data [22] and simulation results). (A) NE levels in the system in the absence (control) and presence (^+^TAN) of neutrophils, and DNase treatment (^+^TAN^+^D) cases at *t* = 22 *h*. (B) Experimental data from the invasion assay in [22] (left column; 4T1 cancer cells) and computational results from mathematical model (right column). The graph shows the (scaled; %) population of invasive tumor cells at *t* = 22 *h* in the absence (control; blue) and presence (^+^TAN; red shaded) of neutrophils, and DNase treatment (^+^TAN^+^D; yellow dotted) cases. Addition of DNase I reduces the number of invading tumor cells by almost 50%.

A comparison of computational results from the mathematical model with experimental data [22] is shown in Figs 4 and 5. Hereafter, in order to calculate the population of cells (tumor cells, N1 TANs, N2 TANs) and level of chemical variables (CXCL-8, TGF-*β*, NET/NE, DNase, MMPs) at various times in the mathematical model, we integrate the density and concentration over the space: density of tumor cells ($\hat{n}\left(t\right)=\int_{\Omega }n\left(x,t\right)\;dx$), N1 TANs (${\hat{N}}_{1}\left(t\right)=\int_{\Omega }N_{1}\left(x,t\right)\;dx$), N2 TANs (${\hat{N}}_{2}\left(t\right)=\int_{\Omega }N_{2}\left(x,t\right)\;dx$), and concentrations of ECM ($\hat{\rho }\left(t\right)=\int_{S}\rho \left(x,t\right)\;dx$), CXCL-8 ($\hat{C}\left(t\right)=\int_{\Omega }C\left(x,t\right)\;dx$), TGF-*β* ($\hat{G}\left(t\right)=\int_{\Omega }G\left(x,t\right)\;dx$), NET/NE ($\hat{E}\left(t\right)=\int_{\Omega }E\left(x,t\right)\;dx$), DNase I ($\hat{D}\left(t\right)=\int_{\Omega }D\left(x,t\right)\;dx$), and MMPs ($\hat{P}\left(t\right)=\int_{\Omega }P\left(x,t\right)\;dx$). In the experiments, Park *et al*. [22] found that the presence of neutrophils in the lower chamber could enhance tumor cell invasion through NET formation and NE activities, and the DNase treatment abrogated the invasion-promoting effect of neutrophils in the lower chamber. Fig 4A–4C shows time courses of the tumor population, population of invasive tumor cells, and neutrophil population (N1 (red solid), N2 (blue dashed) TANs), respectively, in the absence (control) and presence (^+^TAN) of neutrophils. In the presence of neutrophils, both total (Fig 4A) and invasive (Fig 4B) tumor cell populations are increased relative to the control case due to neutrophil transition (Fig 4C) and NE activities (Fig 3D) in the system. After 22*h* the number of 4T1 tumor cells invading the lower chamber almost doubled (∼190%) in the co-culture with neutrophils (red bar (^+^TAN); left panel in Fig 4D) in the lower chamber as compared to the control (blue bar; left panel in Fig 4D) in experiments [22]. In the model simulations, the number of invading tumor cells increased (∼2-fold) in the presence of neutrophils in the lower chamber (red bar (^+^TAN); right panel in Fig 4D) relative to the control (absence of neutrophil (blue bar); right panel in Fig 4D). As Park *et al*. [22] note, several tumor cell lines (4T1, BT-549) invade the lower chamber even in the absence of neutrophils in the lower well, which indicates the intrinsic invasiveness of tumor cells.

In Fig 5, we investigate the effect of DNase I against NET/NE on tumor invasion. Our mathematical model predicts that injection of DNase I in the lower chamber of the transwell can inhibit NET/NE activities (Fig 5A) and reduce the TAN-induced invasiveness of tumor cells (right panel in Fig 5B). Park *et al*. [22] showed that NE inhibition or digestion of the DNA of the NETs by DNase I can effectively abrogated the invasion-promoting effect of TANs in the lower chamber, *i.e*., the number of invasive 4T1 tumor cells was reduced in the presence of the neutralizing DNase I (^+^TAN^+^D) when compared to the TAN case in the absence of the DNase I (^+^TAN) (left panel in Fig 5B). Thus, simulations are in good agreement with experimental data [22]. By definition, NETs are associated with neutrophil proteases with the extracellular histone-bound DNA [88]. In the experiments [22], pro-invasive effects of NETs were shown to be associated with protease activities of NET-associated protease, NE. Park *et al*. [22] found that the NE inhibitor reduced the extension of cancer cell-induced NETs and inhibit TAN’s ability to promote the invasion of 4T1 and BT-549 breast cancer cells. They also found that DNase I treatment can also prevent lung metastasis in mice. However, it is worth observing that this DNase I is not enough to completely inhibit the aggressive migration of tumor cells from the upper chamber to the lower chamber, since they are able to invade in the absence of neutrophils.

**Fig 6:**
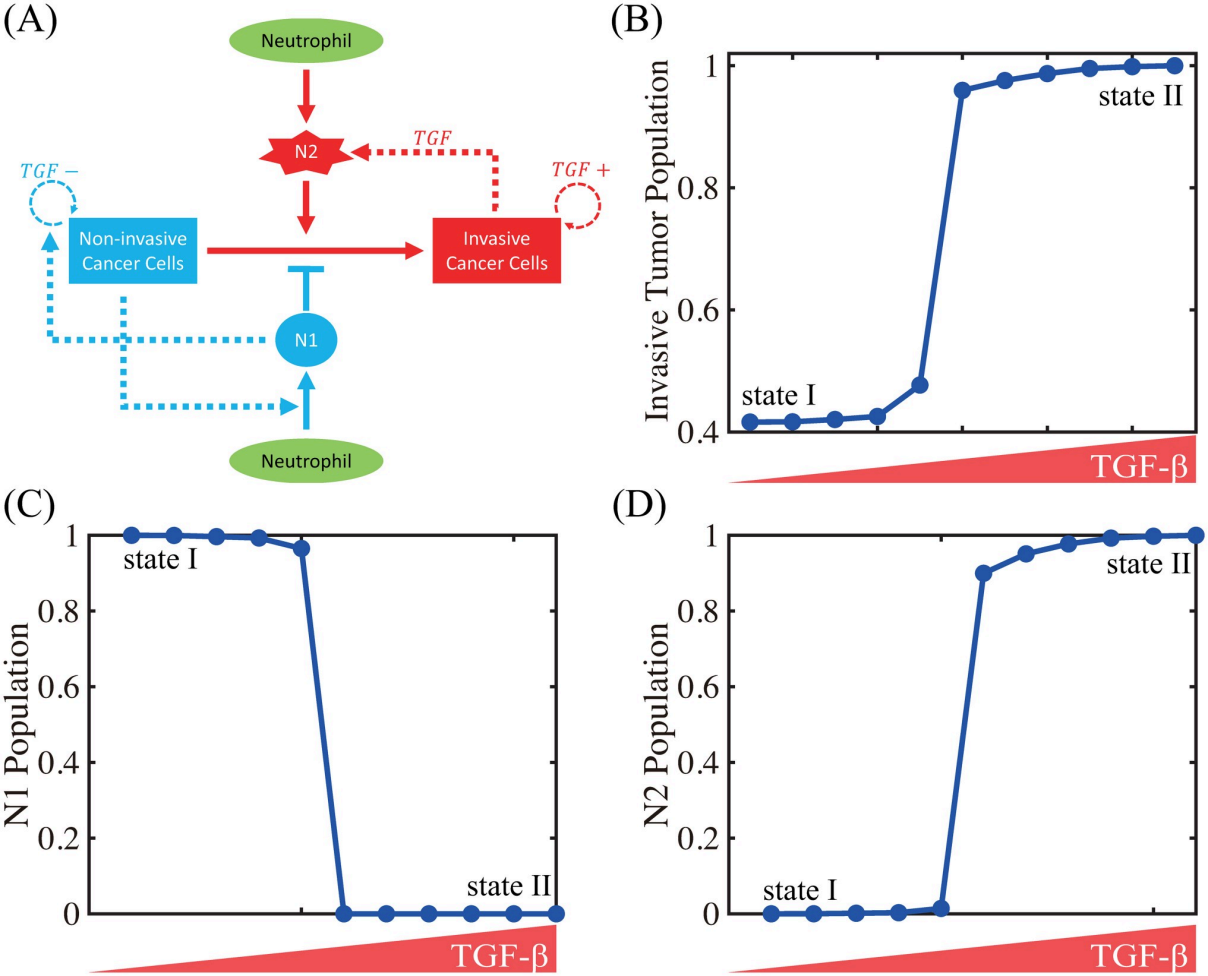
TGF-*β*-mediated cancer-TAN interplay can induce two types of phenotypic states: invasive and non-invasive types. (A) Conceptual interaction network for the mathematical model. (B) Population of invasive tumor cells in the lower chamber as a function of TGF-*β*. (C,D) Populations of N1 (C) and N2 (D) neutrophils as a function of TGF-*β*.

In Fig 6, we illustrate the TGF-*β*-mediated transition between N1 and N2 neutrophils, thus giving rise to two phenotypic states: (a) state I, dominated by non-invasive cancer cells, and (b) state II, dominated by invasive cancer cells. A conceptual schematic of cancer-immune interplay is shown in Fig 6A. Fig 6B–6D shows the scaled populations of invasive cancer cells and, N1 and N2 neutrophils for various levels of TGF-*β* (0, 0.0001, 0.001, 0.002, 0.01, 5, 10, 20, 50, 100, 200). When the TGF-*β* level is low, N1 neutrophils dominate the tumor microenvironment (Fig 6C and 6D), leading to non-invasive states of cancer cells (State I, Fig 6B). As the TGF-*β* level is increased, the N1-dominant system transits to the N2-dominant state (Fig 6C and 6D), resulting in the invasive state of cancer cells (State II, Fig 6B). These results illustrate that the positive feedback loop between N2 neutrophils and invasive phenotype via up-regulation of TGF-*β*, chemotaxis through CXCL8 and NET activities essentially determines the phenotypic transition between non-invasive and invasive states.

**Fig 7:**
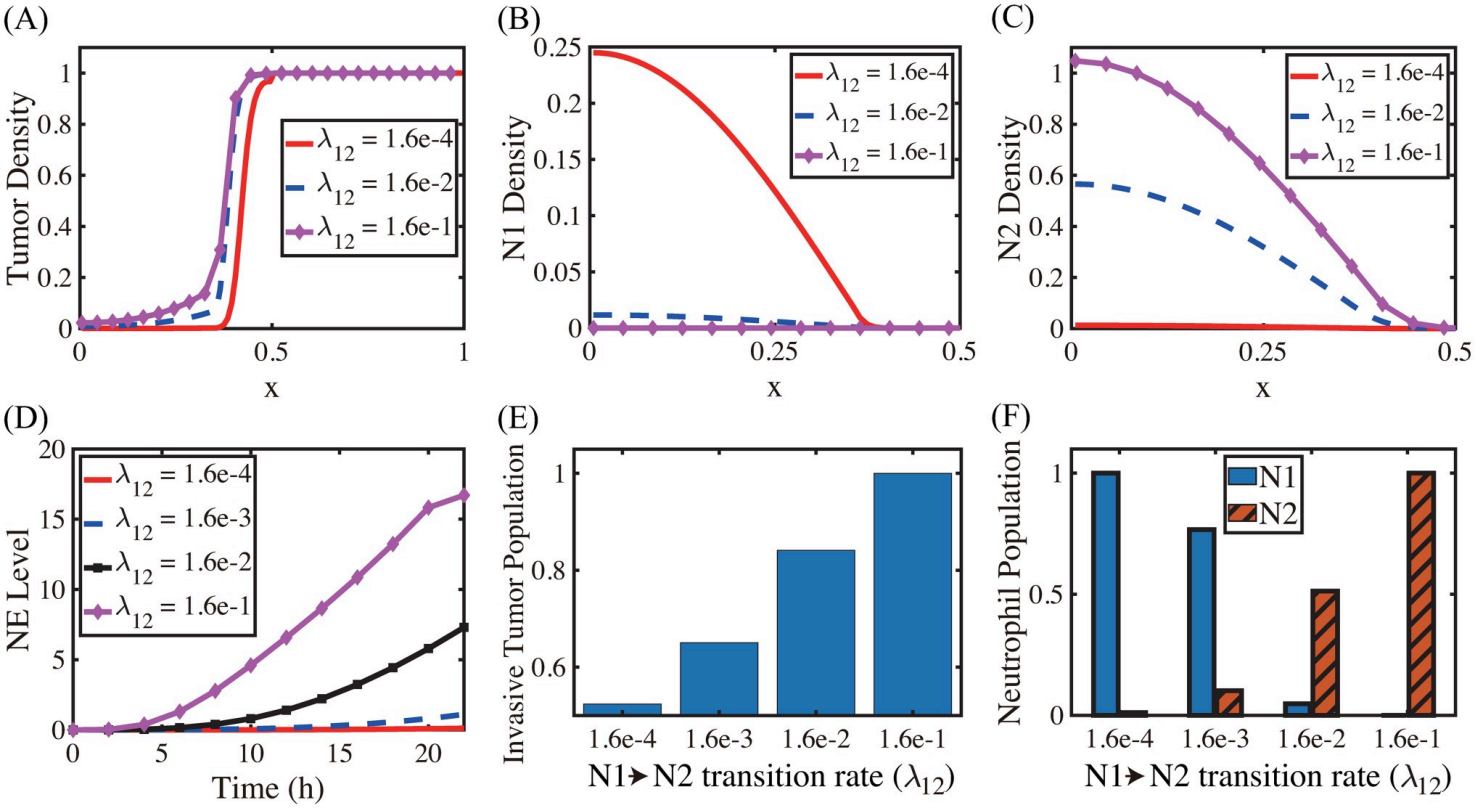
Effect of the N1→N2 transformation on tumor invasion and N1/N2 dynamics. (A) Tumor density profiles on Ω = [0, 1] at the final time (*t* = 22 *h*) for various λ_12_’s (λ_12_ = 1.6 × 10^−4^, 1.6 × 10^−2^, 1.6 × 10^−1^). (B,C) Density profiles of the N1 and N2 TANs in the lower chamber ([0, 0.5]) for the corresponding λ_12_’s in (A). (D) Time courses of NE levels for various values of the differentiation rate (λ_12_ = 1.6 × 10^−4^, 1.6 × 10^−3^, 1.6 × 10^−2^, 1.6 × 10^−1^). (E,F) Scaled population of invasive tumor cells and neutrophils (N1 and N2) at the final time (*t* = 22 *h*) for various λ_12_’s in (D).

The N1→N2 transition of TANs was shown to play a critical role in promoting tumor growth, angiogenesis, invasion [3, 24, 25, 89], and ultimately metastasis initiation [90, 91]. Fig 7A shows the spatial profiles of the tumor cells for various N1→N2 transition rates (λ_12_ = 1.6 × 10^−4^ (red solid), 1.6 × 10^−2^ (blue dashed), 1.6 × 10^−1^ (pink with marks)) at the final time (*t* = 22 *h*). The N1- and N2-dominant spatial profiles of neutrophils in the lower chamber for the corresponding parameter set are shown in Fig 7B and 7C, respectively. If we increase the rate λ_12_ (differentiation degree of anti-tumorigenic TANs to tumor-promoting TANs), the N2 population dominates the lower chamber (Fig 7B and 7C) with the higher population ratio N2:N1 of TANs (Fig 7F). Fig 7D shows time courses of NE levels for various values of the differentiation rate (λ_12_ = 1.6 × 10^−4^, 1.6 × 10^−3^, 1.6 × 10^−2^, 1.6 × 10^−1^). The corresponding populations of invasive tumor cells and neutrophils (N1,N2) at final time (*t* = 22 *h*) are shown in Fig 7E and 7F, respectively. For a larger λ_12_, more aggressive, tumor-promoting N2 TANs in the lower chamber can interact with tumor cells in the upper chamber (Fig 7A) by secreting more NE (Fig 7D). This leads to an increased tumor population and enhanced tumor cell invasion (Fig 7E). This increased invasiveness of the tumor cells is the result of the mutual interactions between tumor cells in the upper well and the neutrophils in the lower well. For instance, the NET/NE level increases as λ_12_ increases (Fig 7D). For a large λ_12_, the most of N1 TANs are converted into the N2 phenotype (4th column (λ_12_ = 1.6 × 10^−1^) in Fig 7F), leading to efficient tumor cell migration (Fig 7E). However, when this transition rate is small (λ_12_ = 1.6 × 10^−4^), the less effective N1 TANs persist in the lower chamber (Fig 7B) with less population of the N2 phenotype (Fig 7C). This results in the slower (or close to zero) production of NE (Fig 7D) by TANs, and lower secretion of both TGF-*β* and MMP by tumor cells, which in turn reduces invasiveness of tumor cells by more than 48% (Fig 7E).

### Application of the model

**Fig 8:**
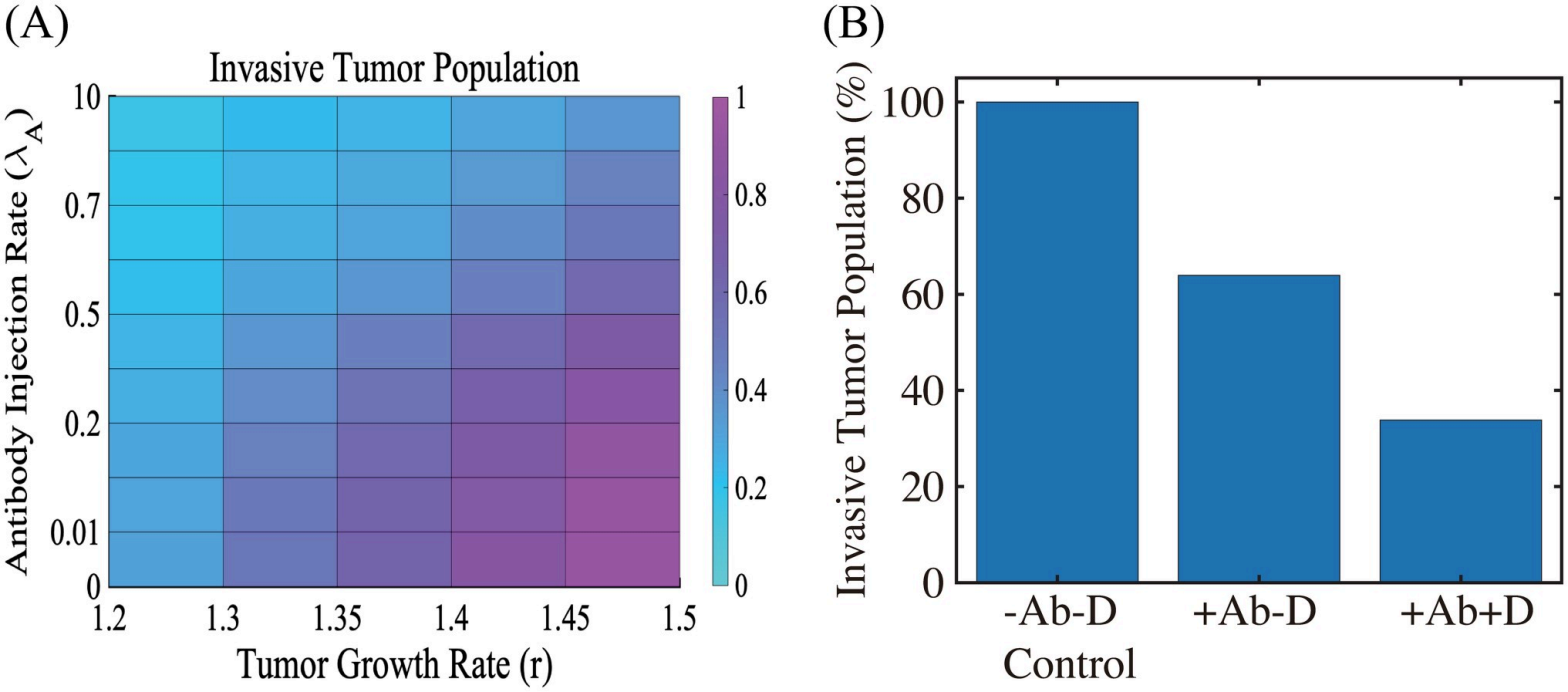
The effect of TGF-*β* blocking (+Ab) and the combined therapy (+Ab+DNase I) on tumor cell invasion. (A) The (relative) population of migrating tumor cells for various growth rates (*r* ∈ [1.2, 1.5]) and injection rates (λ_*A*_ ∈ [0, 10]) of the TGF-*β* antibody. When TGF-*β* activity is inhibited by antibody (*r* = 1.5), fewer cells (62% reduction) invade the lower chamber. (B) Population of migrating tumor cells when the TGF-*β* antibody was added in the absence (+Ab-D) and presence (+Ab+D) of DNase I relative to the control (-Ab-D). When proteolytic activity of tumor cells near the membrane is blocked by DNase I, fewer cells (66% reduction) invade the lower chamber in the presence of TGF-*β* inhibitor.

Blocking TGF-*β* and its receptors was shown to inhibit tumor growth [92, 93] and critical cell invasion [34, 94, 95], decrease tumourigenic potential [92, 96], and reduce metastatic incidence [97] through many different pathways [67, 98]. In order to investigate the effect of TGF-*β* suppression on tumor cell invasion, we introduce a new variable *A*(*x*, *t*) for the TGF-*β* anti-body and derive the following equations including the modified equation of TGF-*β* from Eq (11)

$$\begin{array}{ccc} & & \frac{\partial G}{\partial t}=D_{G}\Delta G+\lambda_{G}n-\mu_{G}G-\mu_{AG}AG, \end{array}$$ (20)

$$\begin{array}{ccc} & & \frac{\partial A}{\partial t}=D_{A}\Delta A+\lambda_{A}-\mu_{A}A, \end{array}$$ (21)

where *μ*_*AG*_ is the consumption rate of TGF-*β* due to antibody reaction, *D*_*A*_ is the diffusion coefficient of the antibody, λ_*A*_ is the injection rate of the antibody, and, finally, *μ*_*A*_ is the natural decay rate of the antibody. Fig 8A shows the (scaled) population of invasive tumor cells for various growth rates (*r* = 1.2, 1.3, 1.35, 1.4, 1.45, 1.5) and injection rates (λ_*A*_ = 0, 1.0 × 10^−2^, 1.0 × 10^−1^, 2.0 × 10^−1^, 3.0 × 10^−1^, 5.0 × 10^−1^, 6.0 × 10^−1^, 7.0 × 10^−1^, 1.0, 1.0 × 10^1^) of the TGF-*β* antibody. For a fixed growth rate of tumor cells, the TGF-*β* inhibitor treatment can effectively reduce the invasiveness of tumor cells. For example, the invasive tumor population with a high dose of antibody (λ_*A*_ = 10) is reduced by 62% compared to the case without the antibody (λ_*A*_ = 0) when *r* = 1.5. However, the increasing growth rate can abrogate this antibody-induced reduction in tumor cell invasion for a fixed λ_*A*_ (*r* = 1.2 → 1.3 → ⋯ → 1.5). DNase treatment, the NE inhibitor, was shown to reduce the NE-mediated invasion of tumor cells (4T1 and BT-549) experimentally [22] and theoretically (Fig 5). Thus, another effective therapeutic approach to slowing tumor invasion is to apply DNase I for a combination therapy (TGF-*β* inhibitor +DNase I) given its pivotal roles in tumorigenesis [4, 8, 22]. We test the efficacy of the combination therapy in Fig 8B. The invasive tumor population is reduced by 36% in response to a high dose of the TGF-*β* inhibitor (λ_*A*_ = 10; +Ab-D in Fig 8B) compared to control (-Ab-D in Fig 8B). Our simulation shows that blocking NE-mediated proteolytic activity of tumor cells near the membrane by DNase I in the presence of TGF-*β* inhibitor can further reduce invasiveness of tumor cells (66% reduction) (Fig 8B).

**Fig 9:**
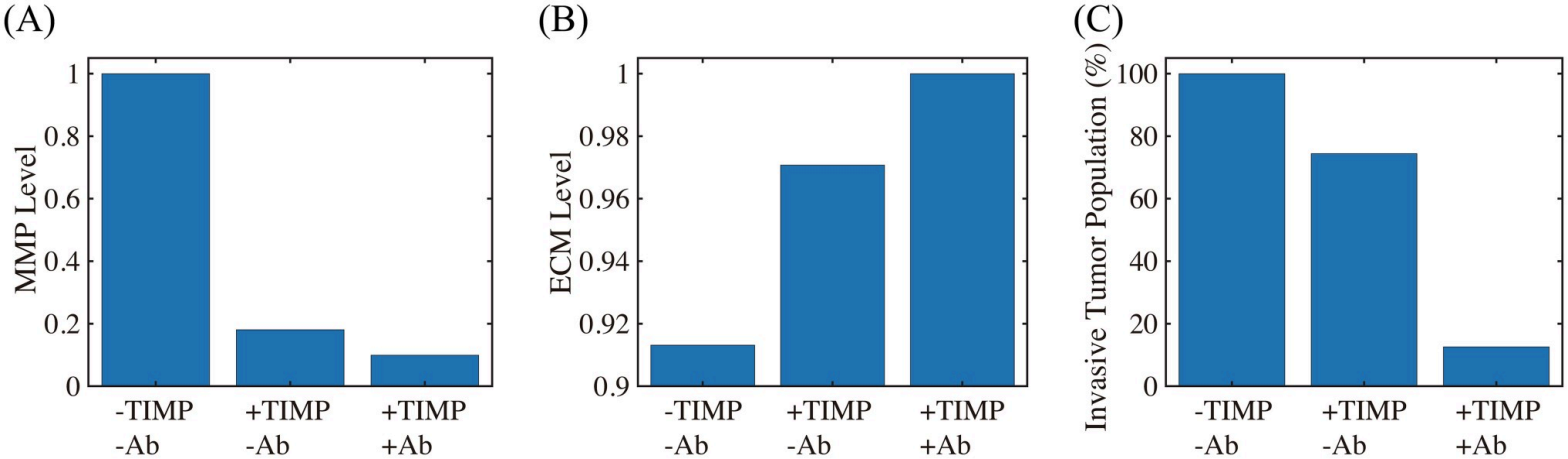
The effect of MMP blocking (+TIMP-Ab) and combined therapy (+TIMP+Ab). (A,B) Levels of MMPs and ECM when MMP activity was blocked by TIMP in the absence (+TIMP-Ab) and presence (+TIMP+Ab) of the TGF-*β* antibody relative to the control (-TIMP-Ab). (C) Population of invading tumor cells corresponding to control (-TIMP-Ab), TIMP treatment (+TIMP-Ab), and combined therapy (+TIMP+Ab), respectively.

Next, we tested if MMP inhibition by TIMP can effectively reduce the TAN-mediated invasion through the transfilter. It has been shown that TIMP treatment can significantly reduce (> 50%) the number of migratory breast cancer cells through 8-*μm* pores in a Boyden invasion chamber assay [99]. In the mathematical model, inhibition of MMP is implemented by injecting TIMP (λ_*M*_ > 0) in Eq (15) which abrogates MMP production through a term of degradation of MMPs, $-\mu_{PM}\frac{PM^{m}}{K_{M}^{m}+M^{m}}$, on the right hand side of Eq (14). We also tested if a combination therapy (TIMP+TGF-*β* inhibitor) can further reduce the invasion potential of tumor cells. Fig 9 shows the (relative) MMP levels, ECM levels, and number of migratory tumor cells at final time (*t* = 22 *h*) for control (-TIMP-Ab), TIMP alone (+TIMP-Ab), and combination treatment (+TIMP+Ab). One can see that TIMP treatment can inhibit the tumor cell motility by 26% (+TIMP-Ab in Fig 9C) through the significant reduction in MMP activities (82%; Fig 9A) and relatively intact ECM level (Fig 9B). An introduction of the TGF-*β* antibody to the system in addition to TIMP (+TIMP+Ab) significantly reduces the tumor cell migration through the filter (> 87%). Blocking tumor-TAN interactions by TGF-*β* inhibitor effectively lowered MMP levels (Fig 9A) and induced the intact ECM levels on the membrane (Fig 9B) [94, 97], contributing to this anti-invasion effect.

**Fig 10:**
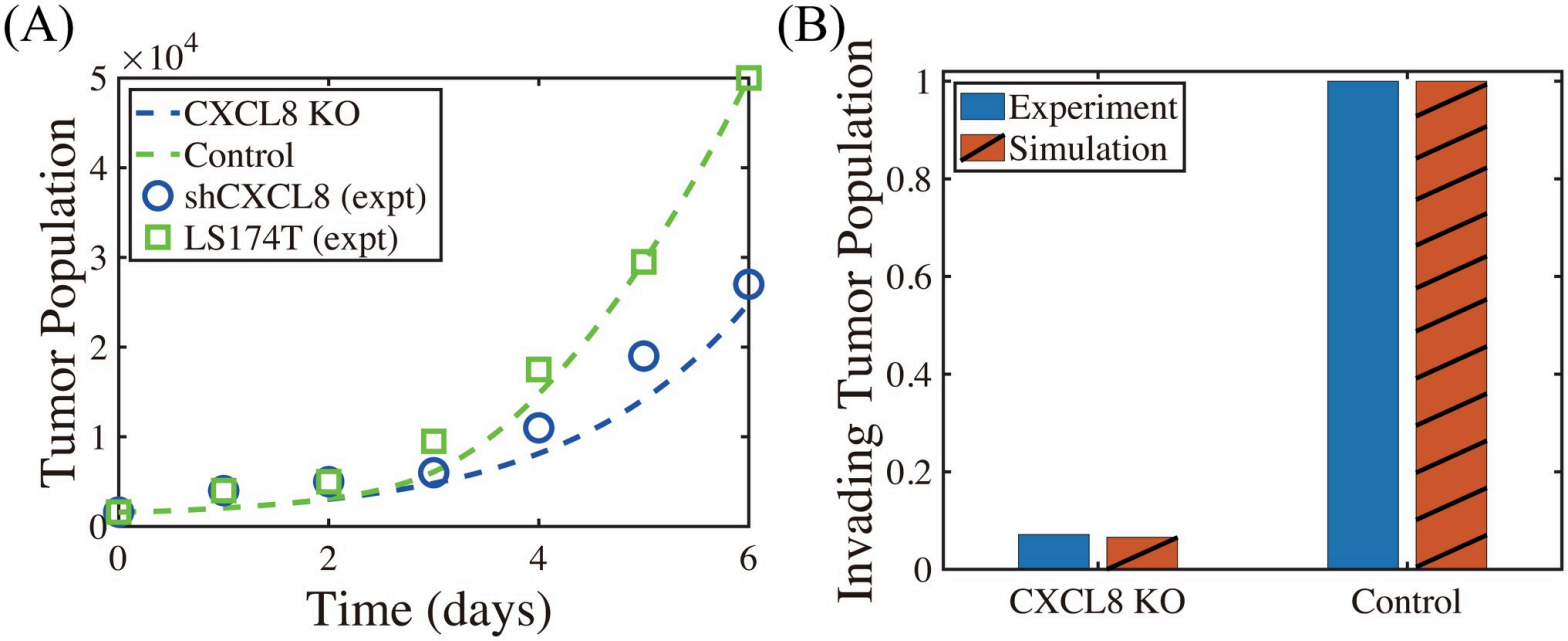
Inhibition of CXCL8 reduces proliferation, viability and invasion (Experiments vs simulation results). (A) Time course of cell proliferation shows a drastic decrease in the LS174T cell population with CXCL8 knockdown (shCXCL8; blue) compared to control (LS174T) in *in vivo* experiments [100]. Model simulation shows a consistent, significant reduction of the tumor cell proliferation in the CXCL knockdown case (CXCL8-KO; blue) compared to control, abrogating tumor growth. (B) CXCL8 knockdown significantly decreases the invasiveness of LS174T cells (blue) [100], as our model simulation illustrates (red).

We now investigate the effect of CXCL-8 on tumor growth and invasion in Fig 10. In our model, CXCL-8 knockdown leads to critical reduction of chemotactic movement of the neutrophils, which in turn reduces tumor growth (blue curve; CXCL8 KO, Fig 10A) and invasion (red; CXCL8 KO, Fig 10B) compared to control due to decreased activities of NE (∼40%) and TGF-*β* (∼50%). These CXCL-KO-mediated reductions in invasive and proliferative potential of tumor cells were consistently observed in experiments. Kumar *et al*. [100] showed that inhibition of CXCL-8 leads to a drastic decrease (∼14-fold) in proliferation of LS174T cells (shCXCL8 in Fig 10A) and significant abrogation of tumor cell invasion (blue in Fig 10B) relative control.

## Discussion

TME plays an important role in regulation of tumor immunogenicity [101], tumor progression, invasion, and metastasis [3, 102]. In particular, the neutrophil-tumor interaction was shown to promote tumor cell invasion, increasing metastatic potential of cancer cells [31]. The invasion and metastasis processes of lung cancer cells may depend on many factors in tumor microenvironment, including immune cells and their cytokines and chemokines [103, 104]. Therefore, targeting players in tumor microenvironment including tumor-associated neutrophils [105] as a therapeutic approach has become more and more important [101, 106]. Experimental [22] and model simulations (Fig 4) suggest that the aggressive tumor invasion through the filter can be promoted by the mutual interaction between tumor cells in the upper chamber and neutrophils in the lower chamber. While the details of the *N*1→*N*2 transition of TANs are still poorly understood, our model consistently suggests the pivotal role of TANs in promoting tumor cell invasion *in vitro* (Figs 6 and 7) through chemotaxis (S4 Text) and haptotaxis (S4 Text). TAN-induced signaling pathways of NET and NE were shown to actively induce tumor invasion and metastasis [71, 107, 108]. On the other hand, the presence of inhibitors of NET/NEs and MMPs was experimentally shown to block tumor invasion [8, 22]. NET was shown to trap the circulating tumor cells (CTCs) in a lung carcinoma model, promoting tumor cell metastasis [19] by up-regulation of *β*_1_-integrin on NETs and cancer cells [109]. Thus, blocking this TAN-assisted tumor invasion can be a critical step to decrease the metastatic potential of the tumor cells. For example, DNase I treatment led to the down-regulation of NE and NET activities and reduced the invasive and metastatic potential of tumor cells (Fig 5), which is in good agreement with experimental studies [22]. Interestingly, impeding the formation of NET by DNase I treatment was shown to halt the actuation of dormant cancer cells [110].

The role of MMPs in regulation of cancer cell invasion and metastasis is well-known [48, 74]. MMP2, for instance, can not only induce tumor cell invasion by degrading ECM but promote tumor cell proliferation by enhancing vessel maturation and function in brain tumors [74]. In particular, inhibition of MMP activity can block cancer cell invasion by suppressing cell-ECM tractions and inducing cell softening [48]. In our model, TIMPs were able to partially block tumor cell invasion by heavy degradation of ECM (Fig 9A). However, when we apply combined therapeutic strategies by TIMP and TGF-*β* antibody, this effectively inhibited the tumor cell invasion through the filter (>87% reduction; Fig 9C). Recently, it was shown that the invasiveness of cancer cells was regulated by MMP catalytic activity via modulation of integrins-FAK signaling network [48]. Recently, TANs are shown to facilitate the metastasis to liver by increased binding activity of CCDC25 to NET DNA [111, 112]. It would be interesting to investigate the detailed mechanism of this signaling.

There are many factors that may change permeability of the narrow intercellular space for cellular infiltration. Even though the permeability parameters are fixed in *in vitro* experiments, the permeability through the narrow gap for cell invasion varies in the *in vivo* system and is regulated by cells and their cytokines/chemokines, changing the invasive potential (S4 Text). For example, TANs and NET can mediate cancer cell extravasation through TAN-CTC adhesion and breaking endothelial cell (EC) barriers, leading to active metastasis [113]. The whole process includes an initial strong adhesion process between a CTC and TANs involving selectins/integrins/ICAM1, and a series of signaling networks for CTC-EC adhesion, increased permeability from physical contraction of ECs, and the final extravasation [104, 113, 114]. A multi-scale model [115] may explain the fundamental mechanism behind this complex process in more detail by taking into account specific cell-cell adhesion [116], ECM-cell interaction [117], mechanical stress [81, 93, 95], fluid flow [118], and intracellular signaling of cellular process [38, 93].

Many studies showed that neutrophil to lymphocyte ratio (NLR) in blood can be an important prognostic factor of cancer progression [119–122] including lung cancers [123, 124]. Our simulation results suggest that the presence of a larger portion of TANs in a TME (or high NLR in blood) can effectively stimulate tumor cell invasion and increase the metastatic potential through increased mutual interaction between tumor cells and N2 TANs (S4 Text). TGF-*β* mediates this critical *N*1 → *N*2 transition of TANs and promotes tumor growth and invasion through the filter (Fig 6) by the phenotypic switch from non-invasive status to invasive status. Therefore, the early detection of initial recruitment of TANs to the TME, for example by calculating NLR, may be an important step in decreasing metastatic potential in patients [8, 28, 122, 125]. Further studies on specific downstream pathways of this CCDC25-ILK-*β*-Parvin signalling [112] would be needed for development of anti-metastatic drugs that target and block the NET-cancer interaction.

Blocking TGF-*β* and its receptors was suggested to a therapeutic approach due to their ability to inhibit tumor growth [92, 93] and critical cell invasion [34, 94, 95], decrease tumourigenic potential [92, 96], and reduce metastatic dissemination [97] through various different pathways including the SMADs family [67, 98]. We showed that inhibition of TGF-*β* can effectively decrease migration potential of tumor cells through the transfilter by reducing the critical interaction between neutrophils and tumor cells (Figs 6 and 8). Model simulation and experiments [100] consistently show that CXCL8 knockdown can reduce tumor growth and invasion (Fig 10). These results illustrate the critical role of neutrophils in tumor cell invasion and importance of inhibition of key players such as TGF-*β* and CXCL8 in suppressing cell infiltration and metastasis potential.

A combined approach with TGF-*β* inhibitor and DNase I can further reduce the invasiveness of tumor cells through the filter (Fig 8). We note, however, that TGF-*β* treatment, not TGF-*β* inhibition, can enhance anti-tumor efficacy through temporal immune suppression in other approaches. For example, Han *et al*. [126] showed that pretreatment with TGF-*β* prior to oncolytic virus (OV) therapy effectively inhibited tumor growth by suppressing resident microglia and natural killer (NK) cells in glioblastoma therapy trial. In the same vein, Kim *et al*. [39] also experimentally and theoretically showed that physical deletion of resident NK cells (^−^*NK*) in the TME, unexpectedly, induced better anti-tumor efficacy relative to the control case in the OV-bortezomib combination therapy since residential NK cells, if not removed, also killed infected tumor cells and depletion of NK cells significantly increased OV-mediated tumor killing. It would be interesting to see how this TGF-*β*-mediated immune suppression affects the tumor invasion and metastasis processes in these combination therapies.

We plan to investigate the role of the continuous spectrum of the *N*1→*N*2 transition and the role of TANs in the regulation of NET-mediated metastasis in future work. Signaling between cells is an integral process in controlling invasive and metastatic potential of tumor cells due to unexpected mutations and chromosomal changes. This signaling often involves indirect communications between various immune cells and spatially-separated tumor cells in the TME. For example, the detailed communication between a tumor at a distant site and neutrophils in bone marrow is poorly understood. Intra-tumor heterogeneity from packing density of various cells in TME and large anisotropic transport through the tissue can affect the signaling pathways [127], thus cancer treatment [128], but despite its importance, corresponding experimental data on signaling are insufficient. Interestingly, the aging TME was recently suggested to influence tumor progression including the critical tumor cell invasion [61]. Thus, *in silico* studies on the effects of these critical interactions on cancer cell invasion, and on the responsiveness of the predictions to physical parameters, can shed insights into guiding experiments aimed at the development of new therapeutic strategies.

## Supporting information

S1 Text Detailed interaction between TANs and tumor cells. (PDF)

S2 Text Nondimensionalization of the mathematical model. (PDF)

S3 Text Parameter estimation of the mathematical model. (PDF)

S4 Text Detailed analysis of the mathematical model. (PDF)
