# Supplementary material for: Role of neutrophil extracellular traps in regulation of lung cancer invasion and metastasis: Structural insights from a computational model
Source: PLoS Comput Biol. 2021 Feb 17;17(2):e1008257. doi: 10.1371/journal.pcbi.1008257 (PMC7920364; doi:10.1371/journal.pcbi.1008257)
Supplement: S1 Text — (PDF) [file pcbi.1008257.s001.pdf]

Supporting Information

Junho Lee, Donggu Lee, Sean Lawler, Yangjin Kim

S1: Detailed interaction between TANs and tumor cells

Lung cancer is still the leading cause of cancer-associated deaths worldwide, with 1.8 million deaths in 2018 [1, 2]. Various cell types such as immune cells, fibroblasts, and endothelial cells in a tumor microenvironment (TME) interact with tumor cells via the cytokines and growth factors. Tumor-associated neutrophils (TANs) are of particular interest because experimental studies showed that they can contribute to the tumor growth, critical invasion, epithelial-mesenchymal transition (EMT), and metastasis of cancer cells [3, 4]. Until recently, neutrophils have been considered as merely a bystander in the TME and metastasis [5–7] but they are emerging as an important player due to consistent and continuous evidences of their tumor-promoting roles [3]. The high neutrophil to lymphocyte ratio (NLR) is considered to be an important indicator of poor prognosis of various cancers [3] including lung cancers [8, 9]. It was shown that cancer cells can secrete CXC chemokines attracting neutrophils to TME [10] and neutrophil invasion is highly correlated with poor clinical outcomes [11, 12]. Neutrophils are the most abundant leukocytes in blood that play important roles in our innate immune system by killing harmful microorganisms via three mechanisms [13]: (i) phagocytosis (engulfing and digestion of bacteria or fungi), (ii) degranulation of cytotoxic enzymes, (iii) neutrophil extracellular traps (NETs), which consist of DNA meshes with cytotoxic enzymes and trap microorganisms in the extracellular space. Therefore, the classical form of neutrophils, called N1 TAN, can induce lysis of tumor cells [14–16] through many mechanisms including reactive oxygen species (ROS)-mediated hypochlorous acid [17, 18] and kill tumor cells via TNF-α [19]. Furthermore, TANs induced from interferons (IFNs) can secrete tumour necrosis factor-related apoptosis-inducing ligand (TRAIL) which then binds to a receptor of tumor cells and passes it to downstream pathways for tumor cell apoptosis [20]. However, tumor-supporting TANs are recruited from tumor cells at a distant TME and infiltrate tumor tissue, leading to tumor growth, invasion, metastasis [21–25] and ultimately, poor clinical outcomes in many cancers [26]. Metastatic cancer cells were able to induce neutrophils to form metastasis-promoting NETs without involving infection processes [27].

While transforming growth factor (TGF-β) is known to facilitate tumor growth, invasion, and metastasis of many types of cancers [28, 29] including breast [30–32] and lung [28] cancers, the tumor-secreted TGF-β was shown to transform N1 TANs (tumor-suppressive phenotype) to N2 TANs (tumor-promoting phenotype) [33–35]. The reverse action of this transformation can be mediated by type I IFN [19, 33], which can inhibit tumor growth [36] through regulation of TYK2, JAK1, STAT family, and their downstream pathways [37].

Excess activity of neutrophil elastase (NE or ELANE) has been shown to cause tissue damage and to harmful remodeling process in lung diseases such as pneumonia, acute lung injury, and cancer [38]. Importantly, NE was shown to infiltrate the TME [39] and promote tumor growth in lung cancers through the PI3K signaling pathways [10]. Neutrophil can also promote the tumor cell invasion by degrading the extracellular matrix (ECM) and basal membrane with NE [40] and matrix metallopeptidase (MMP) [17, 41, 42]. NE was also suggested to induce EMT and metastasis of cancer cells [4, 41, 42]. For example, NE inhibitors were able to inhibit tumor growth and metastasis [40, 43, 44]. TANs were also suggested to drive angiogenesis in malignancy by secreting MMPs, which subsequently promote VEGF secretion [17]. It was shown that neutrophils can promote the tumor cell invasion in the transwell assay [27, 45] and *in vivo* experiments [27, 46, 46].

References

1. Baek S, He Y, Allen BG, Buatti JM, Smith BJ, Tong L, et al. Deep Segmentation Networks Predict Survival of Non-Small Cell Lung Cancer. Sci Rep. 2019;9(1):17286.

2. Organization WH. Cancer. World Health Organization; 2018.

3. Shaul ME, Fridlender ZG. Tumour-associated Neutrophils in Patients With Cancer. Nat Rev Clin Oncol. 2019;16(10):601–620.

4. Leach J, Morton JP, Sansom OJ. Neutrophils: Homing in on the myeloid mechanisms of metastasis. Mol Immunol. 2017;pii: S0161-5890(17):30615–6.

5. Shaul ME, Fridlender ZG. Neutrophils as Active Regulators of the Immune System in the Tumor Microenvironment. J Leukoc Biol. 2017;102(2):343–349.

6. Piccard H, Muschel RJ, Opdenakker S. On the dual roles and polarized phenotypes of neutrophils in tumor development and progression. Crit Rev Oncol Hematol. 2012;82(3):296–309.

7. Kim J, Bae JS. Tumor-Associated Macrophages and Neutrophils in Tumor Microenvironment. Mediators Inflamm. 2016;2016:6058147.

8. Maymani H, Hess K, Groisberg R, Hong DS, Naing A, Piha-Paul S, et al. Predicting outcomes in patients with advanced non-small cell lung cancer enrolled in early phaseimmunotherapy trials. Lung Cancer. 2018;120:137–141.

9. Peng B, Wang YH, Liu YM, Ma LX. Prognostic significance of the neutrophil to lymphocyte ratio in patients with non-small cell lung cancer: a systemic review and meta-analysis. Int J Clin Exp Med. 2015;8(3):3098–106.

10. Houghton AM, Rzymkiewicz DM, Ji H, Gregory AD, Egea EE, Metz HE, et al. Neutrophil elastase-mediated degradation of IRS-1 accelerates lung tumor growth. Nat Med. 2010;16(2):219–223.

11. Bellocq A, Antoine M, Flahault A, Philippe C, Crestani B, Bernaudin JF, et al. Neutrophil alveolitis in bronchioloalveolar carcinoma: induction by tumor-derived interleukin-8 and relation to clinical outcome. Am J Pathol. 1998;152(1):83–92.

12. Foekens JA, Ries C, Look MP, Gippner-Steppert C, Klijn JG, Jochum M. The prognostic value of polymorphonuclear leukocyte elastase in patients with primary breast cancer. Cancer Res. 2003;63(2):337–41.

13. Branzk N, V VP. Molecular mechanisms regulating NETosis in infection and disease. Semin Immunopathol. 2013;35(4):513–30.

14. Singel KK, Segal BH. Neutrophils in the tumor microenvironment: trying to heal the wound that cannot heal. Immunol Rev. 2016;273(1):329–43.

15. Lichtenstein A, Kahle J. Anti-tumor effect of inflammatory neutrophils: characteristics of in vivo generation and in vitro tumor cell lysis. Int J Cancer. 1985;35(1):121–7.

16. Otten MA, Rudolph E, Dechant M, Tuk CW, Reijmers RM, Beelen RH, et al. Immature neutrophils mediate tumor cell killing via IgA but not IgG Fc receptors. J Immunol. 2005;174(9):5472–80.

17. Grecian R, Whyte MKB, Walmsley SR. The role of neutrophils in cancer. Br Med Bull. 2018;xx(xx):xx.

18. Dallegri F, Ottonello L, Ballestrero A, Dapino P, Ferrando F, Patrone F, et al. Tumor cell lysis by activated human neutrophils: analysis of neutrophil-delivered oxidative attack and role of leukocyte function-associated antigen 1. Inflammation. 1991;15(1):15–30.

19. Andzinski L, Kasnitz N, Stahnke S, Wu CF, Gereke M, von Kockritz-Blickwede M, et al. Type I IFNs induce anti-tumor polarization of tumor associated neutrophils in mice and human. Int J Cancer. 2016;138:1982–93.

20. Tecchio C, Huber V, Scapini P, Clzetti F, Margotto D, Todeschini G, et al. IFNalpha-stimulated neutrophils and monocytes release a soluble form of TNF-related apoptosis-inducing ligand (TRAIL/Apo-2 ligand) displaying apoptotic activity on leukemic cells. Blood. 2004;103(10):3837–44.

21. Spiegel A, Brooks MW, Houshyar S, Reinhardt F, Ardolino M, Fessler E, et al. Neutrophils Suppress Intraluminal NK Cell-Mediated Tumor Cell Clearance and Enhance Extravasation of Disseminated Carcinoma Cells. Cancer Discov. 2016;6(6):630–49.

22. Wculek SK, Malanchi I. Neutrophils support lung colonization of metastasis-initiating breast cancer cells. Nature. 2015;528(7582):413–417.

23. Coffelt SB, Kersten K, Doornebal CW, Weiden J, Vrijland K, Hau CS, et al. IL-17-producing $\gamma\delta$ T cells and neutrophils conspire to promote breast cancer metastasis. Nature. 2015;522(7556):345–348.

24. Cools-Lartigue J, Spicer J, McDonald B, Gowing S, Chow S, Giannias B, et al. Neutrophil extracellular traps sequester circulating tumor cells and promote metastasis. J Clin Invest. 2013;123(8):3446–3458.

25. Tazawa H, Okada F, Kobayashi T, Tada M, Mori Y, Une Y, et al. Infiltration of neutrophils is required for acquisition of metastatic phenotype of benign murine fibrosarcoma cells: implication of inflammation-associated carcinogenesis and tumor progression. Am J Pathol. 2003;163(6):2221–32.

26. Shaul ME, Levy L, Sun J, Mishalian I, Singhal S, Kapoor V, et al. Tumor-associated neutrophils display a distinct N1 profile following TGFbeta modulation: A transcriptomics analysis of pro- vs. antitumor TANs. Oncoimmunology. 2016;5(11).

27. Park J, Wysocki RW, Amoozgar Z, Maiorino L, Fein MR, Jorns J, et al. Cancer cells induce metastasis-supporting neutrophil extracellular DNA traps. Sci Transl Med. 2016;8(361):361ra138.

28. Moore-Smith LD, Isayeva T, Lee JH, Frost A, Ponnazhagan S. Silencing of TGF-β1 in tumor cells impacts MMP-9 in tumor microenvironment. Sci Rep. 2017;7(1):8678.

29. Bellomo C, Caja L, Moustakas A. Transforming growth factor β as regulator of cancer stemness and metastasis. Br J Cancer. 2016;115(7):761–9.

30. Kim Y, Wallace J, Li F, Ostrowski M, Friedman A. Transformed epithelial cells and fibroblasts/myofibroblasts interaction in breast tumor: a mathematical model and experiments. J Math Biol. 2010;61(3):401–421.

31. Kim Y, Friedman A. Interaction of tumor with its microenvironment : A Mathematical Model. Bull Math Biol. 2010;72(5):1029–1068.

32. Kim Y, Stolarska M, Othmer HG. The role of the microenvironment in tumor growth and invasion. Prog Biophys Mol Biol. 2011;106:353–379.

33. Shaul ME, Fridlender ZG. Cancer related circulating and tumor-associated neutrophils - subtypes, sources and function. FEBS J. 2018;Epub ahead of print.

34. Saha S, Biswas SK. Tumor-Associated Neutrophils Show Phenotypic and Functional Divergence in Human Lung Cancer. Cancer Cell. 2016;30(1):11–13.

35. Fridlender ZG, Sun J, Kim S, Kapoor V, Cheng G, Ling L, et al. Polarization of tumor-associated neutrophil phenotype by TGF-beta: N1 versus N2 TAN. Cancer Cell. 2009;16(3):183 – 194. doi:10.1016/j.ccr.2009.06.017.

36. Patel MR, Jacobson BA, Ji Y, Drees J, Tang S, Xiong K, et al. Vesicular stomatitis virus expressing interferon-β is oncolytic and promotes antitumor immune responses in a syngeneic murine model of non-small cell lung cancer. Oncotarget. 2015;6(32):33165–77.

37. Takaoka A, Hayakawa S, Yanai H, Stoiber D, Negishi H, Kikuchi H, et al. Integration of interferon-alpha/beta signalling to p53 responses in tumour suppression and antiviral defence. Nature. 2003;424(6948):516–23.

38. Polverino E, Rosales-Mayor E, Dale GE, Dembowsky K, Torres A. The Role of Neutrophil Elastase Inhibitors in Lung Diseases. Chest. 2017;152(2):249 – 262. doi:https://doi.org/10.1016/j.chest.2017.03.056.

39. Manfroi B, Moreaux J, Righini C, Ghiringhelli F, Sturm N, Huard B. Tumor-associated neutrophils correlate with poor prognosis in diffuse large B-cell lymphoma patients. Blood Cancer J. 2018;8(7):66.

40. Sun Z, Yang P. Role of imbalance between neutrophil elastase and alpha 1-antitrypsin in cancer development and progression. Lancet Oncol. 2004;5(3):182–90.

41. Kristensen JH, Karsdal MA, Sand JM, Willumsen N, Diefenbach C, Svensson B, et al. Serological assessment of neutrophil elastase activity on elastin during lung ECM remodeling. BMC Pulm Med. 2015;15:53.

42. Moroy G, Alix AJ, Sapi J, Hornebeck W, Bourguet E. Neutrophil elastase as a target in lung cancer. Anticancer Agents Med Chem. 2012;12(6):565–79.

43. Doi K, Horiuchi T, Uchinami M, Tabo T, Kimura N, Yokomachi J, et al. Neutrophil elastase inhibitor reduces hepatic metastases induced by ischaemia-reperfusion in rats. Eur J Surg. 2002;168:507–510.

44. Inada M, Yamashita J, Nakano S, Ogawa M. Complete inhibition of spontaneous pulmonary metastasis of human lung carcinoma cell line EBC-1 by a neutrophil elastase inhibitor (ONO-5046.Na). Anticancer Res. 1998;18:885–890.

45. Welch DR, Schissel DJ, Howrey RP, Aeed PA. Tumor-elicited polymorphonuclear cells, in contrast to normal circulating polymorphonuclear cells, stimulate invasive and metastatic potentials of rat mammary adenocarcinoma cells. Proc Natl Acad Sci U S A. 1989;86(15):5859–63.

46. Slattery MJ, Dong C. Neutrophils influence melanoma adhesion and migration under flow conditions. Int J Cancer. 2003;106(5):713–722.
