# Supplementary material for: Role of neutrophil extracellular traps in regulation of lung cancer invasion and metastasis: Structural insights from a computational model
Source: PLoS Comput Biol. 2021 Feb 17;17(2):e1008257. doi: 10.1371/journal.pcbi.1008257 (PMC7920364; doi:10.1371/journal.pcbi.1008257)
Supplement: S2 Text — (PDF) [file pcbi.1008257.s002.pdf]

Supporting Information

Junho Lee, Donggu Lee, Sean Lawler, Yangjin Kim

S2: Nondimensionalization of the mathematical model

The governing equations for the variables in the main text in the presence of TGF-β antibody (A) are as follows:

$$
\begin{aligned}
\frac{\partial n}{\partial t} &= \nabla \cdot \left(D_n \nabla n - \chi_E n \frac{\nabla E}{\delta_E + \sigma_E |\nabla E|} - \chi_\rho I_S n \frac{\nabla \rho}{\delta_\rho + \sigma_\rho |\nabla \rho|} \right) \\
&\quad + r\left(1 + r_E \frac{E^m}{k_E^m + E^m}\right) n \left(1 - \frac{n}{n_0}\right) - \mu_n N_1 n \quad \text{in } \Omega_*,\ t > 0, \\
\frac{\partial N_1}{\partial t} &= \nabla \cdot \left(D_1 \nabla N_1 - \chi_1 N_1 \frac{\nabla C}{\delta_1 + \sigma_C |\nabla C|} \right) + \lambda_1 N_1 - \lambda_{12} G N_1 \quad \text{in } \Omega_*,\ t > 0, \\
\frac{\partial N_2}{\partial t} &= \nabla \cdot \left(D_2 \nabla N_2 - \chi_2 N_2 \frac{\nabla C}{\delta_2 + \sigma_C |\nabla C|} \right) + \lambda_{12} G N_1 + \lambda_2(G) N_2 \quad \text{in } \Omega_*,\ t > 0, \\
\frac{d\rho}{dt} &= -(\mu_{\rho 1} E + \mu_{\rho 2} P) n \quad \text{in } S,\ t > 0, \\
\frac{\partial C}{\partial t} &= D_C \Delta C + \lambda_C n - \mu_C C \quad \text{in } \Omega_*,\ t > 0, \\
\frac{\partial G}{\partial t} &= D_G \Delta G + \lambda_G n - \mu_G G - \mu_{AG} A G \quad \text{in } \Omega_*,\ t > 0, \\
\frac{\partial E}{\partial t} &= D_E \Delta E + \lambda_E N_2 - \mu_E E - \mu_{ED} \frac{E D^l}{K_D^l + D^l} \quad \text{in } \Omega_*,\ t > 0, \\
\frac{\partial P}{\partial t} &= D_P \Delta P + \lambda_P N_2 - \mu_{PM} \frac{P M^m}{K_M^m + M^m} - \mu_P P \quad \text{in } \Omega_*,\ t > 0, \\
\frac{\partial D}{\partial t} &= D_D \Delta D + \lambda_D I_{\Omega_I} - \mu_D D \quad \text{in } \Omega_*,\ t > 0, \\
\frac{\partial M}{\partial t} &= D_M \Delta M + \lambda_M - \mu_M M \quad \text{in } \Omega_*,\ t > 0, \\
\frac{\partial A}{\partial t} &= D_A \Delta A + \lambda_A - \mu_A A \quad \text{in } \Omega_*,\ t > 0.
\end{aligned}
$$

We non-dimensionalize the variables and the parameters in the governing equations above as follows

$$
\begin{aligned}
&\bar{t} = \frac{t}{T},\ \bar{x} = \frac{x}{L},\ \bar{n} = \frac{n}{n^*},\ \overline{N}_1 = \frac{N_1}{N_1^*},\ \overline{N}_2 = \frac{N_2}{N_2^*},\ \bar{\rho} = \frac{\rho}{\rho^*},\ \overline{C} = \frac{C}{C^*},\ \overline{G} = \frac{G}{G^*},\ \overline{E} = \frac{E}{E^*}, \overline{P} = \frac{P}{P^*}, \\
&\overline{D} = \frac{D}{D^*},\ \overline{M} = \frac{M}{M^*},\ \overline{A} = \frac{A}{A^*},\ \overline{D}_n = \frac{D_n}{D_\dagger},\ \overline{D}_1 = \frac{D_1}{D_\dagger},\ \overline{D}_2 = \frac{D_2}{D_\dagger},\ \overline{D}_C = \frac{D_C}{D_\dagger},\ \overline{D}_G = \frac{D_G}{D_\dagger}, \\
&\overline{D}_E = \frac{D_E}{D_\dagger},\ \overline{D}_P = \frac{D_P}{D_\dagger},\ \overline{D}_D = \frac{D_D}{D_\dagger},\ \overline{D}_M = \frac{D_M}{D_\dagger},\ \overline{D}_A = \frac{D_A}{D_\dagger},\ \bar{r} = rT,\ \bar{r}_E = r_E,\ \bar{k}_E = \frac{k_E}{E^*}, \\
&\bar{n}_0 = \frac{n_0}{n^*},\ \bar{\lambda}_1 = \lambda_1 T,\ \bar{\lambda}_{12} = \lambda_{12} T G^*,\ \bar{\lambda}_{12}^\dagger = \frac{\lambda_{12} T G^* N_1^*}{N_2^*},\ \bar{\lambda}_2 = \lambda_2 T,\ \bar{\lambda}_C = \frac{\lambda_C T n^*}{C^*},\ \bar{\lambda}_G = \frac{\lambda_G T n^*}{G^*}, \\
&\bar{\lambda}_E = \frac{\lambda_E T N_2^*}{E^*},\ \bar{\lambda}_P = \frac{\lambda_P T N_2^*}{P^*},\ \bar{\lambda}_D = \frac{\lambda_D T}{D^*},\ \bar{\lambda}_M = \frac{\lambda_M T}{M^*},\ \bar{\lambda}_A = \frac{\lambda_A T}{A^*},\ \bar{\mu}_n = \mu_n T N_1^*,
\end{aligned}
$$

Table S1. Reference variables used in the model.

	Description	Dimensional Value	Refs.
T	Time	$1\ h$	
L	Length	$1.0\ mm$	
$D_\dagger$	Diffusion coefficients ($= L^2/T$)	$2.78 \times 10^{-6}\ cm^2 s^{-1}$	
n^*	Tumor density	$2.5{\times}10^4\ cells/cm^3$	[1, 2]
N_1^*	N1 neutrophil density	$1.0{\times}10^5\ cells/cm^3$	[1, 3, 4]
N_2^*	N2 neutrophil density	$= N_1^*$	[1, 3]
ρ^*	ECM density	$5.0{\times}10^{-4} g/cm^3$	[5–10]
C^*	CXCL8 (IL-8) concentration	$1{\times}10^{-12}\ g/cm^3$	[11]
G^*	TGF-β concentration	$1.1\ {\times}10^{-8}\ g/cm^3$	[2, 12, 13]
E^*	Neutrophil elastase concentration	$6.46\ {\times}10^{-9}\ g/cm^3$	[14, 15]
P^*	MMP concentration	$1.6{\times}10^{-9}\ g/cm^3$	[16]
D^*	NE inhibitor (DNase I) concentration	$3.2{\times}10^{-9}\ g/cm^3$	[17–19]
M^*	TIMP concentration	$4.6385\ {\times}10^{-8}\ g/cm^3$	[20]
A^*	Concentration of anti-body of TGF-β	$0.172\ \mu M$	[21]

$$\overline{\mu}_{\rho 1} = \frac{\mu_{\rho 1} T E^* n^*}{\rho^*},\ \overline{\mu}_{\rho 2} = \frac{\mu_{\rho 2} T P^* n^*}{\rho^*},\ \overline{\mu}_C = \mu_C T,\ \overline{\mu}_G = \mu_G T,\ \overline{\mu}_E = \mu_E T,\ \overline{\mu}_{ED} = \mu_{ED} T,$$

$$\overline{K}_D = \frac{K_D}{D^*},\ \overline{\mu}_P = \mu_P T,\ \overline{\mu}_D = \mu_D T,\ \overline{K}_M = \frac{K_M}{M^*},\ \overline{\mu}_M = \mu_M T,\ \overline{\mu}_{PM} = \mu_{PM} T,\ \overline{\mu}_A = \mu_A T,$$

$$\overline{\mu}_{AG} = \mu_{AG} T A^*,\ \overline{\chi}_E = \frac{\chi_E T}{L},\ \overline{\delta}_E = \frac{\delta_E L}{E^*},\ \overline{\sigma}_E = \sigma_E,\ \overline{\chi}_\rho = \frac{\chi_\rho T}{L},\ \overline{\delta}_\rho = \frac{\delta_\rho L}{\rho^*},\ \overline{\sigma}_\rho = \sigma_\rho,$$

$$\overline{\chi}_1 = \frac{\chi_1 T}{L},\ \overline{\delta}_1 = \frac{\delta_1 L}{C^*},\ \overline{\sigma}_C = \sigma_C,\ \overline{\chi}_2 = \frac{\chi_2 T}{L},\ \overline{\delta}_2 = \frac{\delta_2 L}{C^*}.$$

where reference values $T, L, D, n^*, N_1^*, N_2^*, \rho^*, C^*, G^*, E^*, P^*, D^*, M^*, A^*$ are given in Table S1. Here, $D_\dagger = L^2/T$ is the characteristic diffusion coefficient for characteristic length L and time T in Table S1.

Then, the governing equations in a dimensionless form are

$$
\begin{aligned}
\frac{\partial \overline{n}}{\partial \overline{t}} &= \overline{\nabla} \cdot (\overline{D}_n \overline{\nabla}\,\overline{n}) - \overline{\nabla} \cdot \left(\overline{\chi}_E \; \overline{n} \; \frac{\overline{\nabla E}}{\overline{\delta}_E + \overline{\sigma}_E |\overline{\nabla E}|} \right) - \overline{\nabla} \cdot \left(\overline{\chi}_\rho \; I_{\overline{S}} \overline{n} \; \frac{\overline{\nabla}\overline{\rho}}{\overline{\delta}_\rho + \overline{\sigma}_\rho |\overline{\nabla}\overline{\rho}|} \right) \\
&\quad + \overline{r} \left(1 + \overline{r}_E \frac{\overline{E}^m}{\overline{k}_E^m + \overline{E}^m} \right) \overline{n} \left(1 - \frac{\overline{n}}{\overline{n}_0} \right) - \overline{\mu}_n \overline{N}_1 \overline{n} \quad \text{in } \overline{\Omega}_*, \; \overline{t} > 0, \\
\frac{\partial \overline{N}_1}{\partial \overline{t}} &= \overline{\nabla} \cdot \left(\overline{D}_1 \overline{\nabla N}_1 - \overline{\chi}_1 \overline{N}_1 \frac{\overline{\nabla C}}{\overline{\delta}_1 + \overline{\sigma}_C |\overline{\nabla C}|} \right) + \overline{\lambda}_1 \overline{N}_1 - \overline{\lambda}_{12} \overline{G} \overline{N}_1 \quad \text{in } \overline{\Omega}_*, \; \overline{t} > 0, \\
\frac{\partial \overline{N}_2}{\partial \overline{t}} &= \overline{\nabla} \cdot \left(\overline{D}_2 \overline{\nabla N}_2 - \overline{\chi}_2 \overline{N}_2 \frac{\overline{\nabla C}}{\overline{\delta}_2 + \overline{\sigma}_C |\overline{\nabla C}|} \right) + \overline{\lambda}_{12} \overline{G} \overline{N}_1 + \overline{\lambda}_2(\overline{G}) \overline{N}_2 \quad \text{in } \overline{\Omega}_*, \; \overline{t} > 0, \\
\frac{\overline{d\rho}}{\overline{dt}} &= -(\overline{\mu}_{\rho 1} \overline{E} + \overline{\mu}_{\rho 2} \overline{P}) \overline{n} \qquad \text{in } \overline{S}, \; t > 0, \\
\frac{\partial \overline{C}}{\partial \overline{t}} &= \overline{D}_C \overline{\Delta C} + \overline{\lambda}_C \overline{n} - \overline{\mu}_C \overline{C} \qquad \text{in } \overline{\Omega}_*, \; \overline{t} > 0, \\
\frac{\partial \overline{G}}{\partial \overline{t}} &= \overline{D}_G \overline{\Delta G} + \overline{\lambda}_G \overline{n} - \overline{\mu}_G \overline{G} - \overline{\mu}_{AG} \overline{AG} \qquad \text{in } \overline{\Omega}_*, \; \overline{t} > 0, \\
\frac{\partial \overline{E}}{\partial \overline{t}} &= \overline{D}_E \overline{\Delta E} + \overline{\lambda}_E \overline{N}_2 - \overline{\mu}_E \overline{E} - \overline{\mu}_{ED} \frac{\overline{E}\; \overline{D}^l}{\overline{K}_D^l + \overline{D}^l} \qquad \text{in } \overline{\Omega}_*, \; \overline{t} > 0, \\
\frac{\partial \overline{P}}{\partial \overline{t}} &= \overline{D}_P \overline{\Delta P} + \overline{\lambda}_P \overline{N}_2 - \overline{\mu}_{PM} \frac{\overline{P}\; \overline{M}^m}{\overline{K}_M^m + \overline{M}^m} - \overline{\mu}_P \overline{P} \qquad \text{in } \overline{\Omega}_*, \; \overline{t} > 0, \\
\frac{\partial \overline{D}}{\partial \overline{t}} &= \overline{D}_D \overline{\Delta D} + \overline{\lambda}_D I_{\Omega_I} - \overline{\mu}_D \overline{D} \qquad \text{in } \overline{\Omega}_*, \; \overline{t} > 0, \\
\frac{\partial \overline{M}}{\partial \overline{t}} &= \overline{D}_M \overline{\Delta M} + \overline{\lambda}_M - \overline{\mu}_M \overline{M} \qquad \text{in } \overline{\Omega}_*, \; \overline{t} > 0, \\
\frac{\partial \overline{A}}{\partial \overline{t}} &= \overline{D}_A \overline{\Delta A} + \overline{\lambda}_A - \overline{\mu}_A \overline{A} \qquad \text{in } \overline{\Omega}_*, \; \overline{t} > 0.
\end{aligned}
$$

Note that $\overline{\lambda}_{12}^\dagger = \overline{\lambda}_{12}$ under the assumption of $N_1^* = N_2^*$; otherwise $\overline{\lambda}_{12}^\dagger$ is different from $\overline{\lambda}_{12}$.

References

1. Park J, Wysocki RW, Amoozgar Z, Maiorino L, Fein MR, Jorns J, et al. Cancer cells induce metastasis-supporting neutrophil extracellular DNA traps. Sci Transl Med. 2016;8(361):361ra138.

2. Kim Y, Wallace J, Li F, Ostrowski M, Friedman A. Transformed epithelial cells and fibroblasts/myofibroblasts interaction in breast tumor: a mathematical model and experiments. J Math Biol. 2010;61(3):401–421.

3. Moghe PV, Nelson RD, Tranquillo RT. Cytokine-stimulated chemotaxis of human neutrophils in a 3-D conjoined fibrin gel assay. J Immunol Methods. 1995;180(2):193–211.

4. Cools-Lartigue J, Spicer J, McDonald B, Gowing S, Chow S, Giannias B, et al. Neutrophil extracellular traps sequester circulating tumor cells and promote metastasis. J Clin Invest. 2013;123(8):3446–3458.

5. Perumpanani AJ, Byrne HM. Extracellular matrix concentration exerts selection pressure on invasive cells. Eur J Cancer. 1999;35(8):1274–80.

6. Namy P, Ohayon J, Tracqui P. Critical conditions for pattern formation and in vitro tubulogenesis driven by cellular traction fields. J Theor Biol. 2004;227(1):103–20.

7. Delvoye P, Wiliquet P, Leveque JL, Nusgens BV, Lapiere CM. Measurement of mechanical forces generated by skin fibroblasts embedded in a three-dimensional collagen gel. J Invest Dermatol. 1991;97(5):898–902.

8. Vernon RB, Angello JC, Iruela-Arispe ML, et al. Reorganization of basement membrane matrices by cellular traction promotes the formation of cellular networks in vitro. Lab Invest. 1992;66:536–547.

9. Vailhe B, Ronot X, Tracqui P, Usson Y, Tranqui L. In vitro angiogenesis is modulated by the mechanical properties of fibrin gels and is related to alpha v beta3 integrin localization. In Vitro Cell Dev Biol-Animal. 1997;33:763–773.

10. Bemis LT, Schedin P. Reproductive state of rat mammary gland stroma modulates human breast cancer cell migration and invasion. Cancer Res. 2000;60(13):3414–8.

11. Ren Y, Poon RT, Tsui HT, Chen WH, Li Z, Lau C, et al. Interleukin-8 serum levels in patients with hepatocellular carcinoma: correlations with clinicopathological features and prognosis. Clin Cancer Res. 2003;9(16 Pt 1):5996–6001.

12. Kunz-Schughart LA, Wenninger S, Neumeier T, Seidl P, Knuechel R. Three-dimensional tissue structure affects sensitivity of fibroblasts to TGF-beta 1. Am J Physiol Cell Physiol. 2003;284(1):C209–19.

13. Kong FM, Anscher MS, Murase T, Abbott BD, Iglehart JD, Jirtle RL. Elevated plasma transforming growth factor-beta 1 levels in breast cancer patients decrease after surgical removal of the tumor. Ann Surg. 1995;222(2):155162.

14. Bellocq A, Antoine M, Flahault A, Philippe C, Crestani B, Bernaudin JF, et al. Neutrophil alveolitis in bronchioloalveolar carcinoma: induction by tumor-derived interleukin-8 and relation to clinical outcome. Am J Pathol. 1998;152(1):83–92.

15. Hind CR, Joyce H, Tennent GA, Pepys MB, Pride NB. Plasma leucocyte elastase concentrations in smokers. J Clin Pathol. 1991;44(3):232–5.

16. Jia Y, Zeng ZZ, Markwart SM, Rockwood KF, Ignatoski KM, Ethier SP, et al. Integrin fibronectin receptors in matrix metalloproteinase-1-dependent invasion by breast cancer and mammary epithelial cells. Cancer Res. 2004;64(23):8674–81.

17. Prince WS, Baker DL, Dodge AH, Ahmed AE, Chestnut RW, Sinicropi DV. Pharmacodynamics of recombinant human DNase I in serum. Clin Exp Immunol. 1998;113(2):289–96.

18. Chitrabamrung S, Bannett JS, Rubin RL, Tan EM. A radial diffusion assay for plasma and serum deoxyribonuclease I. Rheumatol Int. 1981;1(2):49–53.

19. Macanovic M, Lachmann PJ. Measurement of deoxyribonuclease I (DNase) in the serum and urine of systemic lupus erythematosus (SLE)-prone NZB/NZW mice by a new radial enzyme diffusion assay. Clin Exp Immunol. 1997;108(2):220–6.

20. Hire JM, Evanson JL, Johnson PC, Zumbrun SD, Guyton MK, 3rd McPherson JC, et al. Variance of Matrix Metalloproteinase (MMP) and Tissue Inhibitor of Metalloproteinase (TIMP) Concentrations in Activated, Concentrated Platelets From Healthy Male Donors. J Orthop Surg Res. 2014;9:29.

21. Yingling JM, McMillen WT, Yan L, Huang H, Sawyer JS, Graff J, et al. Preclinical Assessment of Galunisertib (LY2157299 Monohydrate), a First-In-Class Transforming Growth factor-β Receptor Type I Inhibitor. Oncotarget. 2017;9(6):6659–6677.
