# Supplementary material for: Role of neutrophil extracellular traps in regulation of lung cancer invasion and metastasis: Structural insights from a computational model
Source: PLoS Comput Biol. 2021 Feb 17;17(2):e1008257. doi: 10.1371/journal.pcbi.1008257 (PMC7920364; doi:10.1371/journal.pcbi.1008257)
Supplement: S3 Text — (PDF) [file pcbi.1008257.s003.pdf]

Supporting Information

Junho Lee, Donggu Lee, Sean Lawler, Yangjin Kim

S3: Parameter estimation of the mathematical model

Parameters

Dimensional values of the various parameters of the mathematical model are provided in Tables 1 and 2 in the main text.

D_n (Radom motility of tumor cells): Various cell motility values were reported. The cell motility coefficient of typical animal's cells was estimated to be 5.0×10^{-10} $cm^2 s^{-1}$ [1]. The motility constant of EC cells in a medium including angiogenic factor was measured to be 7.0×10^{-9} $cm^2 s^{-1}$ [2]. Burgess *et al.* [3] also adapted the similar value, $D_n = 1.7 \times 10^{-9}$ $cm^2 s^{-1}$. Smaller values of the cell motility constant were used for tumor cells (10^{-10} $cm^2 s^{-1}$ [4]) and glioma cells (1.0×10^{-12} $cm^2 s^{-1}$ [5]). While the experimental measurement for cell motility of human glioma [6] and glioblastoma cells [7] in 2D substrate suggests the value in the range of $(1.16 \times 10^{-10} - 2.31 \times 10^{-9})$ $cm^2 s^{-1}$, Stein *et al.* [8] estimated a higher value of $D_n (=2.31 \times 10^{-8}$ $cm^2 s^{-1})$ based on the experimental observation. In our simulations, we take $D_n = 2.5 \times 10^{-8}$ $cm^2 s^{-1}$.

D_1, D_2 (Radom motility of Neutrophils): In an *in vitro* migration study [9], the random motility constant of human neutrophils was estimated to be in the range of $(2.0 \times 10^{-9} - 1.2 \times 10^{-8})$ $cm^2 s^{-1}$ depending on various IL-8 concentrations ($(1.0 \times 10^{-9} - 1.0 \times 10^{-6})$ M) in a 3-D conjoined fibrin gel assay. We take $D_1 = D_2 = 1.1 \times 10^{-8}$ $cm^2 s^{-1}$.

D_C (Diffusion coefficient of CXCL8): A diffusion constant of the fluorescent CXCL8 (IL-8) was measured and estimated to be 2.54×10^{-6} $cm^2 s^{-1}$ with a small standard error (5.28×10^{-8} $cm^2 s^{-1}$) in a study of CXCL8-induced movement of neutrophils [9], which further used in a study of the role of neutrophils in tumor cell rolling in a shear blood flow [10] and neutrophil chemotaxis model [11]. Based on these studies, we take $D_C = 2.5 \times 10^{-6}$ $cm^2 s^{-1}$.

D_E (Diffusion coefficient of Neutrophil elastase): A diffusion coefficient of human neutrophil elastase was estimate to be $9.54 \times 10^{-7} cm^2 s^{-1}$ [12–15]. We take $D_E = 5.0 \times 10^{-7} cm^2 s^{-1}$ [16, 17].

D_P (Diffusion coefficient of MMPs): In experiments of the diffusive movement of MMP-1 molecules in the collagen fibril, Saffarian *et al.* [18] estimated the diffusion constant of MMP1 to be $(8 \pm 1.5) \times 10^{-9}$ $cm^2 s^{-1}$ for a wild-type and $(6.7 \pm 1.5) \times 10^{-9}$ $cm^2 s^{-1}$ for an inactive form. On the other hand, Sherratt and Murray [19] took the much larger values of the diffusion coefficient in the magnitude of 10^{-7} $cm^2 s^{-1}$. In the study of glioma cell migration, Kim *et al.* [20] estimated the MMP diffusion coefficient to be 8.0×10^{-9} $cm^2 s^{-1}$ in the brain tissue. In our simulation, we take $D_P = 5.0 \times 10^{-10}$ $cm^2 s^{-1}$.

D_D (Diffusion coefficient of DNase I): Chitrabamrung *et al.* investigated the diffusion process of plasma and serum deoxyribonuclease I (DNase I) by a relatively simple radial diffusion technique where the rate of hydrolysis of DNA by bovine pancreatic DNase was measured by means of the diameter changes in time (increasing from 0 to $13mm$ during $50h$) [21]. Based on these values at various DNase concentrations ((10-100) ng/ml), we estimate the diffusion coefficient $D_D = 7.374 \times 10^{-6}$ $cm^2 s^{-1}$.

λ_{12} (TGF-β-mediated transition rate from N1 TANs to N2 TANs): It has been shown that TGF-β mediates the critical transition of TAN's phenotype, a tumor-suppressive (N1) phenotype to a more tumor-promoting (N2) phenotype [22–24]. We assume that the transformation rate of N1 TANs to N2

phenotype far exceeds the proliferation rate of N2 TANs (λ_2). Therefore, by using the reference value of TGF-β, $G^* = 1.1 \times 10^{-8}\ g/cm^3$ [25–27] in Section below, and taking the approximation $\lambda_{12}G^* \approx 35.6\lambda_2$, we get $\lambda_{12} = 4.08 \times 10^3\ cm^3 g^{-1} s^{-1}$.

μ_C (Decay rate of CXCL8): The half-life of IL-8 (CXCL8) is short, less than 4 h [28–30]. We take 3 hours for the half-life, leading to the decay rate, $\mu_C = \frac{ln(2)}{3\ h} = 0.231\ h^{-1} = 6.42 \times 10^{-5}\ s^{-1}$.

μ_E (Decay rate of Neutrophil elastase): The half life of neutrophil elastase was reported to be 12 $\sim$ 30 h in a NE transcriptional study [31] and we take the half life 24 h, leading to $\mu_E = \frac{ln(2)}{24\ h} = 8.02 \times 10^{-6}\ s^{-1}$.

μ_P (Decay rate of MMPs): MMPs are usually localized in the invasion front of a migrating cell or a leading group of invasive tumor cells [32,33] with a high secretion rate and relatively short half-life. By taking the half-life of MMP as 228 minutes [34], we get $\mu_P = \frac{ln(2)}{228\ m} = 5.0 \times 10^{-5}\ s^{-1}$.

μ_D (Decay rate of DNase I): The half-life of neutrophil elastase inhibitors in human hepatocytes was estimated to be over 130 min [35,36]. Sivelestat aminoacetate tetrahydrate, a synthetic human neutrophil elastase inhibitor, has a relatively short half-life of 2 h in the human body [37,38]. The half-life of DNase was reported to be 8 min at pH 8.0 and 2 h at pH 6.7 Calcium [39]. By taking the half life of 120 min, we get $\mu_D = \frac{ln(2)}{120\ min} = 9.627 \times 10^{-5}\ s^{-1}$.

μ_{ED} (Degradation rate of NE by DNase I): DNase activity was enhanced (30-fold increase) for the rhDNase concentration in the range of (1-500) ng/ml [40], which dramatically remove DNA components. We take $\mu_{ED} = (2.8 \times 10^{-4}$- $2.8 \times 10^{-2})\ s^{-1}$.

μ_M (Decay rate of TIMPs): The half-life of TIMP is reported to be 42.2 $hours$ in blood [41], leading to the decay rate $\mu_M = \frac{ln(2)}{42.2\ h} = 4.56 \times 10^{-6}\ s^{-1}$.

μ_A (Decay rate of an anti-body): The half-life of galunisertib, TGF-β inhibitor, is reported to be 0.26 $hour$ in rat, 0.3 $hour$ in mice, and 2.26 $hour$ in dogs [42]. By estimating the half-life of 3 $hours$ in human [24], we get $\mu_A = \frac{ln(2)}{3\ h} = 6.42 \times 10^{-5}\ s^{-1}$.

Reference values of main variables

C^* (CXCL8 level (IL-8)): Preoperative serum IL-8 was significantly elevated in hepatocellular carcinoma patients (17.6 pg/ml) compared with healthy individuals (1.0 pg/ml) [43]. We take $C^* = 1\ pg/ml$ from the healthy individual.

E^* (NE level): NE levels were much higher in BAC patients as compared with control groups (6.46±2.28 vs 0.93±0.33 ng/ml) [44]. Plasma elastase levels were reported to be 98.7 ng/ml for non-smokers and 131.9 ng/ml for smokers [45]. We take $E^* = 6.46\ ng/ml$.

M^* (TIMP level): TIMP concentrations were reported to be 44.25 ng/ml in young individuals and 48.52 ng/ml in older generations (age > 30) [46]. So we take $M^* = 46.385\ ng/ml$.

A^* (TGF-β antibody): Yingling *et al.* [47] found that Galunisertib, TGF-β anti-body, is a highly selective TGFβR1 inhibitor with a concentration of $\text{IC}_{50} = 0.172\ \mu M$. So we take $A^* = 0.172\ \mu M$.

References

1. Bray D. Cell Movements: From Molecules to Motility. Garland Pub; 2000.

2. Stokes CL, Lauffenburger DA. Analysis of the Roles of Microvessel Endothelial Cell Random Motility and Chemotaxis in Angiogenesis. J theor Biol. 1991;152:377–403.

3. Burgess PK, Kulesa PM, Murray JD, r EC J. The interaction of growth rates and diffusion coefficients in a three-dimensional mathematical model of gliomas. J Neuropathol Exp Neurol. 1997;56(6):704–13.

4. Anderson ARA, Chaplain MAJ. Continuous and Discrete mathematical Models of Tumor-Induced Angiogenesis. Bull Math Biol. 1998;60:857–900.

5. Sander LM, Deisboeck TS. Growth patterns of microscopic brain tumors. Phys Rev E. 2002;66:051901.

6. Demuth T, Hopf NJ, Kempski O, Sauner D, Herr M, Giese A, et al. Migratory activity of human glioma cell lines in vitro assessed by continuous single cell observation. Clin Exp Metastasis. 2000;18(7):589–97.

7. Hegedus B, Zach J, Czirok A, Lovey J, Vicsek T. Irradiation and Taxol treatment result in non-monotonous, dose-dependent changes in the motility of glioblastoma cells. J Neurooncol. 2004;67(1-2):147–57.

8. Stein AM, Demuth T, Mobley D, Berens M, Sander LM. A mathematical model of glioblastoma tumor spheroid invasion in a three-dimensional in vitro experiment. Biophys J. 2007;92(1):356–65.

9. Moghe PV, Nelson RD, Tranquillo RT. Cytokine-stimulated chemotaxis of human neutrophils in a 3-D conjoined fibrin gel assay. J Immunol Methods. 1995;180(2):193–211.

10. Liang S, Hoskins M, Khanna P, Kunz RF, Dong C. Effects of the tumor-leukocyte microenvironment on melanoma-neutrophil adhesion to the endothelium in a shear flow. Cell Mol Bioeng. 2008;1(2-3):189–200.

11. Jeon NL, Baskaran H, Dertinger SK, Whitesides GM, de Water LV, Toner M. Neutrophil chemotaxis in linear and complex gradients of interleukin-8 formed in a microfabricated device. Nat Biotechnol. 2002;20(8):826–30.

12. Campbell EJ, Campbell MA, Boukedes SS, Owen CA. Quantum proteolysis by neutrophils: implications for pulmonary emphysema in alpha 1-antitrypsin deficiency. J Clin Invest. 1999;104(3):337–44.

13. Liou TG, Campbell EJ. Nonisotropic enzyme–inhibitor interactions: a novel nonoxidative mechanism for quantum proteolysis by human neutrophils. Biochemistry. 1995;34(49):16171–7.

14. Baugh RJ, Travis J. Human leukocyte granule elastase: rapid isolation and characterization. Biochemistry. 1976;15(4):836–41.

15. Ohlsson K, Olsson I. The neutral proteases of human granulocytes. Isolation and partial characterization of granulocyte elastases. Eur J Biochem. 1974;42(2):519–27.

16. Aimetti AA, Tibbitt MW, Anseth KS. Human neutrophil elastase responsive delivery from poly(ethylene glycol) hydrogels. Biomacromolecules. 2009;10(6):1484–9.

17. Weber LM, Lopez CG, Anseth KS. Effects of PEG hydrogel crosslinking density on protein diffusion and encapsulated islet survival and function. J Biomed Mater Res A. 2009;90(3):720–9.

18. Saffarian S, Collier IE, Marmer BL, Elson EL, Goldberg G. Interstitial collagenase is a Brownian ratchet driven by proteolysis of collagen. Science. 2004;306(5693):108–11.

19. Sherratt JA, Murray JD. Models of epidermal wound healing. ProcR SocLond. 1990;B241:29–36.

20. Kim Y, Jeon H, Othmer HG. The role of the tumor microenvironment in glioblastoma: A mathematical model. IEEE Trans Biomed Eng. 2017;64(3):519–527.

21. Chitrabamrung S, Bannett JS, Rubin RL, Tan EM. A radial diffusion assay for plasma and serum deoxyribonuclease I. Rheumatol Int. 1981;1(2):49–53.

22. Shaul ME, Fridlender ZG. Tumour-associated Neutrophils in Patients With Cancer. Nat Rev Clin Oncol. 2019;16(10):601–620.

23. Shaul ME, Levy L, Sun J, Mishalian I, Singhal S, Kapoor V, et al. Tumor-associated neutrophils display a distinct N1 profile following TGFbeta modulation: A transcriptomics analysis of pro- vs. antitumor TANs. Oncoimmunology. 2016;5(11).

24. Kim Y, Lee D, Lee J, Lee S, Lawler S. Role of tumor-associated neutrophils in regulation of tumor growth in lung cancer development: A mathematical model. PLoS One. 2019;14(1):e0211041.

25. Kim Y, Wallace J, Li F, Ostrowski M, Friedman A. Transformed epithelial cells and fibroblasts/myofibroblasts interaction in breast tumor: a mathematical model and experiments. J Math Biol. 2010;61(3):401–421.

26. Kunz-Schughart LA, Wenninger S, Neumeier T, Seidl P, Knuechel R. Three-dimensional tissue structure affects sensitivity of fibroblasts to TGF-beta 1. Am J Physiol Cell Physiol. 2003;284(1):C209–19.

27. Kong FM, Anscher MS, Murase T, Abbott BD, Iglehart JD, Jirtle RL. Elevated plasma transforming growth factor-beta 1 levels in breast cancer patients decrease after surgical removal of the tumor. Ann Surg. 1995;222(2):155162.

28. Orlikowsky TW, Neunhoeffer F, Goelz R, Eichner M, Henkel C, Zwirner M, et al. Evaluation of IL-8-concentrations in plasma and lysed EDTA-blood in healthy neonates and those with suspected early onset bacterial infection. Pediatr Res. 2004;56(5):804–9.

29. Redl H, Schlag G, Bahrami S, Schade U, Ceska M, Stutz P. Plasma neutrophil-activating peptide-1/interleukin-8 and neutrophil elastase in a primate bacteremia model. J Infect Dis. 1991;164(2):383–8.

30. Redl H, Schlag G, Bahrami S, Dinges HP, Schade U, Ceska M. Markers of endotoxin related leukocyte activation and injury mechanisms. Prog Clin Biol Res. 1991;367:83–100.

31. Yoshimura K, Crystal RG. Transcriptional and posttranscriptional modulation of human neutrophil elastase gene expression. Blood. 1992;79(10):2733–40.

32. Friedl P, Alexander S. Cancer invasion and the microenvironment: Plasticity and reciprocity. Cell. 2011;147(5):992–1009.

33. Kim Y, Othmer HG. A hybrid model of tumor-stromal interactions in breast cancer. Bull Math Biol. 2013;75:1304–1350.

34. Kim Y, Friedman A. Interaction of tumor with its microenvironment : A Mathematical Model. Bull Math Biol. 2010;72(5):1029–1068.

35. von Nussbaum F, Li VM. Neutrophil elastase inhibitors for the treatment of (cardio)pulmonary diseases: Into clinical testing with pre-adaptive pharmacophores. Bioorg Med Chem Lett. 2015;25(20):4370–81.

36. Gnamm C, Oost T, Peters S. 20140221335 A1. US Patent. 2014;.

37. Yoshikawa N, Inomata T, Okada Y, Shimbo T, Takahashi M, Akita K, et al. Sivelestat sodium hydrate reduces radiation-induced lung injury in mice by inhibiting neutrophil elastase. Mol Med Rep. 2013;7(4):1091–5.

38. Nakashima H, Akimoto A, Kitagawa T, et al. General pharmacological studies of sodium N-[2-[4-(2,2-dimethethyl-propionyloxy) phenylsulfonylamino] benzoyl] aminoacetate tetrahydrate (ONO-5046 Na). Pharmacometrics. 1997;54:267–277.

39. Liao TH, Ho HC, Abe A. Chemical modification of bovine pancreatic deoxyribonuclease with phenylglyoxal–the involvement of Arg-9 and Arg-41 in substrate binding. Biochim Biophys Acta. 1991;1079(3):335–42.

40. Prince WS, Baker DL, Dodge AH, Ahmed AE, Chestnut RW, Sinicropi DV. Pharmacodynamics of recombinant human DNase I in serum. Clin Exp Immunol. 1998;113(2):289–96.

41. Sa Y, Hao J, Samineni D, Clark J, Pyne-Geithman G, Broderick J, et al. Brain Distribution and Elimination of Recombinant Human TIMP-1 After Cerebral Ischemia and Reperfusion in Rats. Neurol Res. 2011;33(4):433–8.

42. Herbertz S, Sawyer JS, Stauber AJ, Gueorguieva I, Driscoll KE, Estrem ST, et al. Clinical development of galunisertib (LY2157299 monohydrate), a small molecule inhibitor of transforming growth factor-beta signaling pathway. Drug Design, Development and Therapy. 2015;9:4479–4499.

43. Ren Y, Poon RT, Tsui HT, Chen WH, Li Z, Lau C, et al. Interleukin-8 serum levels in patients with hepatocellular carcinoma: correlations with clinicopathological features and prognosis. Clin Cancer Res. 2003;9(16 Pt 1):5996–6001.

44. Bellocq A, Antoine M, Flahault A, Philippe C, Crestani B, Bernaudin JF, et al. Neutrophil alveolitis in bronchioloalveolar carcinoma: induction by tumor-derived interleukin-8 and relation to clinical outcome. Am J Pathol. 1998;152(1):83–92.

45. Hind CR, Joyce H, Tennent GA, Pepys MB, Pride NB. Plasma leucocyte elastase concentrations in smokers. J Clin Pathol. 1991;44(3):232–5.

46. Hire JM, Evanson JL, Johnson PC, Zumbrun SD, Guyton MK, 3rd McPherson JC, et al. Variance of Matrix Metalloproteinase (MMP) and Tissue Inhibitor of Metalloproteinase (TIMP) Concentrations in Activated, Concentrated Platelets From Healthy Male Donors. J Orthop Surg Res. 2014;9:29.

47. Yingling JM, McMillen WT, Yan L, Huang H, Sawyer JS, Graff J, et al. Preclinical Assessment of Galunisertib (LY2157299 Monohydrate), a First-In-Class Transforming Growth factor-β Receptor Type I Inhibitor. Oncotarget. 2017;9(6):6659–6677.
