# Supplementary material for: Role of neutrophil extracellular traps in regulation of lung cancer invasion and metastasis: Structural insights from a computational model
Source: PLoS Comput Biol. 2021 Feb 17;17(2):e1008257. doi: 10.1371/journal.pcbi.1008257 (PMC7920364; doi:10.1371/journal.pcbi.1008257)
Supplement: S4 Text — (PDF) [file pcbi.1008257.s004.pdf]

Supporting Information

Junho Lee, Donggu Lee, Sean Lawler, Yangjin Kim

S4: Detailed analysis of the mathematical model

Effect of chemotaxis

We study the effect of chemotaxis of tumor cells on tumor cell infiltration into the lower chamber through the ECM barrier in the middle. Fig S1 shows the tumor density (Fig S1A) and the populations of migratory tumor cells (left panel in Fig S1B) at $t = 22\,h$ as a function of the chemotactic sensitivity ($\chi_E = 4.02 \times 10^{-6}$, 4.02×10^{-5}, 8.02×10^{-5}). One sees that as χ_E increases, the population of migratory tumor cells increases and they move faster toward the ECM membrane. We note that these tumor cells grow faster in the lower chamber due to the higher level of NE and NE-mediated growth, leading to increased total tumor populations (right panel in Fig S1B).

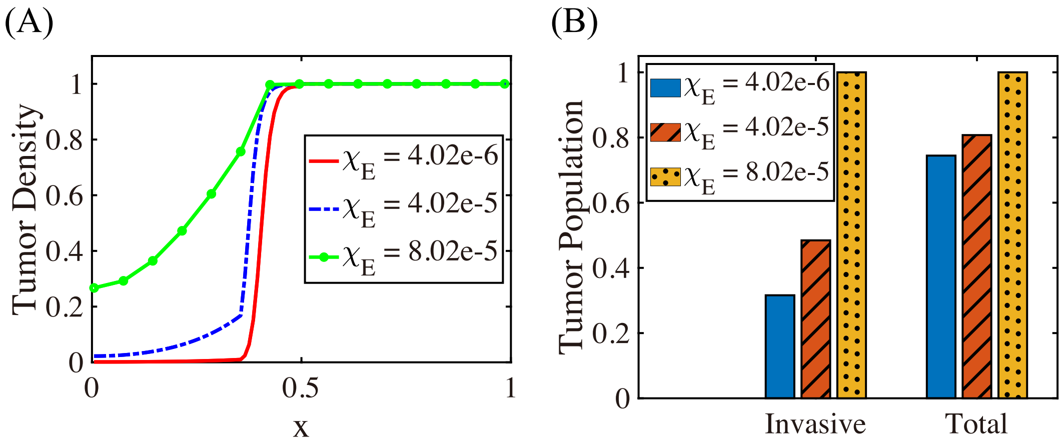

Figure S1. The effect of chemotaxis on tumor cell migration. (A) Spatial profiles of the tumor cell density on $\Omega = [0, 1]$ at final time ($t = 22\ h$) for different values of the chemotactic sensitivity ($\chi_E = 4.02 \times 10^{-6}$, 4.02×10^{-5}, 8.02×10^{-5}). (B) The (scaled) number of invasive (left panel) and total (right panel) tumor cells at final time ($t = 22\ h$) as a function of χ_E. As χ_E increases, the number of migrating tumor cells is increased.

Effect of haptotaxis

Fig S2A shows the spatial profiles of tumor densities at final time ($t = 22\ h$) for various haptotactic parameters ($\chi_\rho = 1.26 \times 10^{-5}$, 1.26×10^{-4}, 3.26×10^{-4}). One can observe that more tumor cells invade the lower chamber when χ_ρ is increased ($\chi_\rho : 1.26 \times 10^{-5} \to 1.26 \times 10^{-4} \to 3.26 \times 10^{-4}$). The scaled numbers of migrated tumor cells at final time ($t = 22\ h$) as a function of the haptotactic sensitivity χ_ρ are shown in Fig S2B. As χ_ρ increases, the number of migrating tumor cells and overall speed of invasion increase. We also explored the combined sensitivity of chemotactic- and haptotactic-stimulus on the invasiveness of tumor populations [1] (data not shown). We, again, found that the tumor cells show higher potential of invading the lower chamber when they are stimulated with higher chemotactic (χ_E) and haptotactic (χ_ρ) sensitivities. Furthermore, the combined movement at final time is more evident in the lower chamber due to the stronger degree of interaction between tumor cells and N2 TANs.

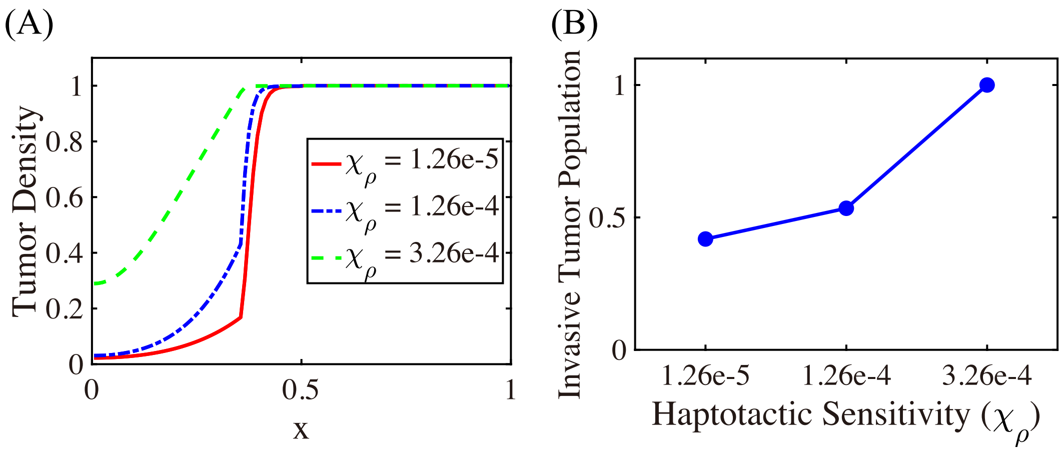

Figure S2. The effect of haptotaxis on invasion. (A) Spatial profiles of the tumor cell density on $\Omega = [0, 1]$ at $t = 22$ h for different values of the haptotactic sensitivity ($\chi_\rho = 1.26 \times 10^{-5}$, 1.26×10^{-4}, 3.26×10^{-4}). (B) The population of migrating tumor cells in the lower chamber as a function of χ_ρ at final time ($t = 22$ h). As the haptotactic parameter (χ_ρ) increases the tumor cells invade into the region occupied by the neutrophils more rapidly.

Effect of permeability of the transfilter

In Fig S3, we investigate the effect of permeability of the transfilter on the number of migrating tumor cells. In our modeling framework, the basic permeability parameters (γ_c for cells and larger value γ ($\gamma_c < \gamma$) for chemicals) represent the relative pore size in the membrane of the transwell for cells and chemicals [2,3]. Two parameters (γ_c, γ) were reduced or increased at the same fold. One observes that as the transfilter pore size increases (or decreases), more (or less) tumor cells in the upper chamber cross the membrane (Fig S3A), increasing (or decreasing) the overall tumor population in the lower chamber and the number of invasive tumor cells (Fig S3B). This is due to the ease of crossing the weakened physical barrier and increased proliferation rate of tumor cells via the enhanced cross-talk between tumor cells and neutrophils in the lower chamber [2].

Effect of TANs

We first test the effect of TANs on invasive and metastatic potential of tumor cells in Fig S4. To quantify the TAN-promoting effect on tumor-cell invasion, we initially placed different TAN populations on the lower chamber with various initial ratios of the TAN population to tumor population (TAN:tumor=1:100,1:10,1:2, 1:1, 2:1, 5:1, 10:1). As the ratio (TAN:tumor) increases, the invasive potential of tumor cells through the filter also increased (Fig S4A) due to the increased population of N2 TANs (Fig S4B) and increased potential of chemotactic- and haptotactic-mediated movement. For example, the up-regulation of NEs (Fig S4C) and MMPs (Fig S4D) promotes the ECM degradation (Fig S4F), increasing the haptotactic potential. On the other hand, the up-regulation of TGF-β (Fig S4E) enhances the critical $N1 \rightarrow N2$ transition, further increasing N2 TAN activities (Fig S4B) and closely connecting the positive feedback loop between the tumor cells in the upper chamber and TANs in the lower chamber. Welch et al [4] tested the effect of tumor-elicited PMNs (tcPMN) on tumor cell invasion by placing both TANs and tumor cells on top of the Matrigel in the upper chamber and found that tcPMN can effectively stimulate invasive and metastatic potentials of mammary adenocarcinoma cells. For example, they found that the invasive potential of tumor cells (MTLn2) increased up to 7-fold and 25.5-fold higher for PMN:tumor ratios of 3:1 and 30:1, respectively [4]. These results suggest that the relative portion

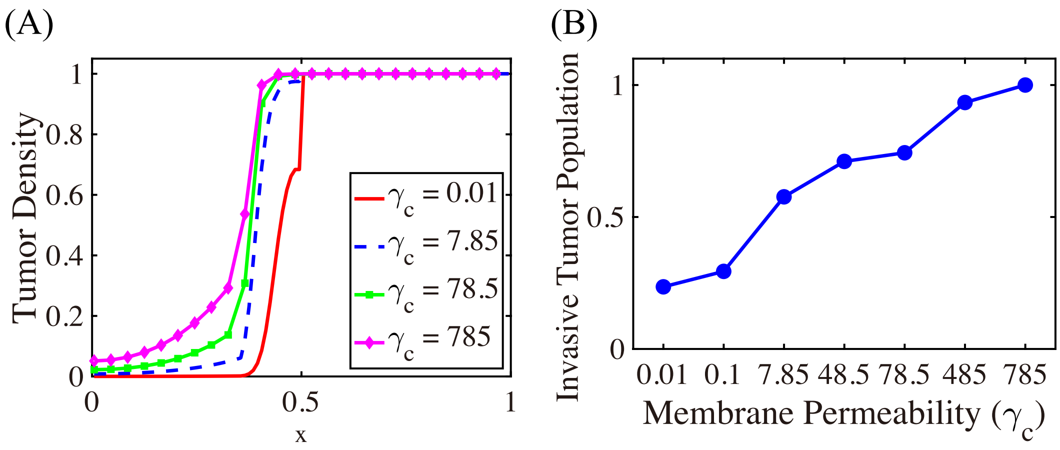

Figure S3. The effect of the transfilter permeability (γ_c, γ) on tumor cell invasion. (A) Spatial profiles of tumor densities at the final time ($t = 22\ h$) for various permeability parameters of the transmembrane ($\gamma_c = 1.0 \times 10^{-2}$, 7.85, 7.85×10^1, 7.85×10^2). (B) The (scaled) number of migratory tumor cells as a function of the permeability ($\gamma_c = 1.0 \times 10^{-2}$, 1.0×10^{-1}, $7.85, 4.85 \times 10^1$, 7.85×10^1, 4.85×10^2, 7.85×10^2).

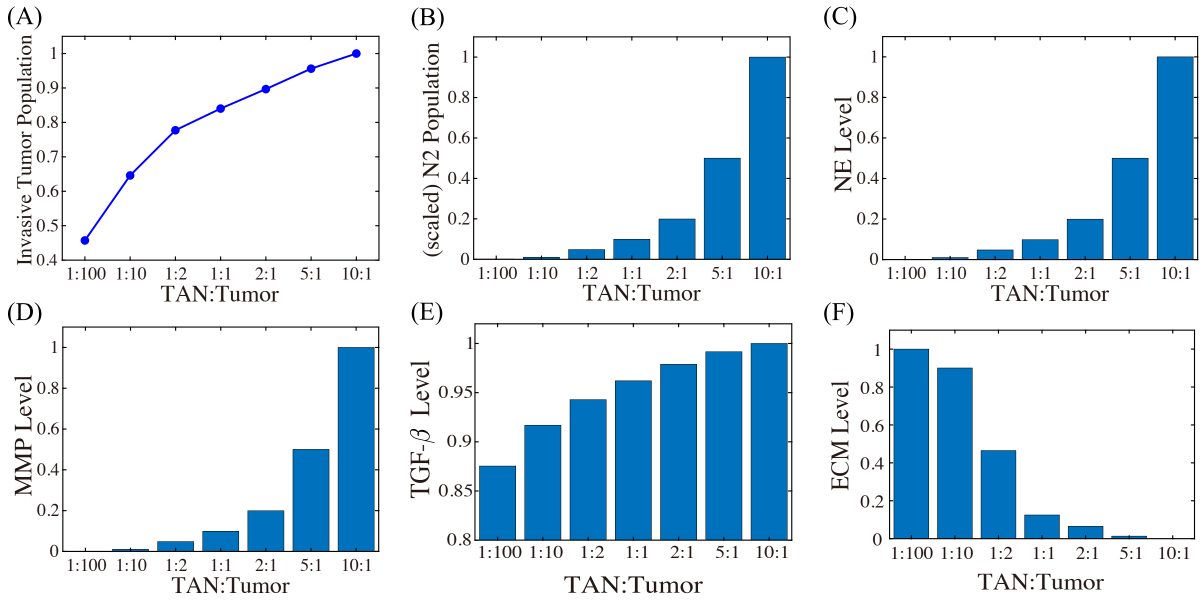

Figure S4. The effect of the TANs on invasive potential of tumor cells. (A) The (scaled) Invasive tumor population at final time $t = 22\ h$ with various initial ratios of the TAN population to tumor population (TAN:tumor=1:100,1:10,1:2, 1:1, 2:1, 5:1, 10:1). (B-F) Scaled N2 population (B), NE levels (C), MMP levels (D), TGF-β levels (E), and ECM levels on the membrane (F) corresponding to the same ratios in (A) at final time.

of TANs in a TME can affect the critical tumor cell invasion, therefore, metastatic potential of tumor cells, and that initial recruitment strength of TANs to the TME by tumor cells may be an important prognostic factor in determining metastatic potential in patients as suggested in experiments [5–8]. In particular, neutrophil-to-lymphocyte ratio (NLR) in blood was shown to be an important prognostic factor for cancer progression [7, 9–11] including lung cancers [12, 13].

Sensitivity Analysis

The mathematical model developed in this work includes 44 parameters, some of which are available in the literature or can be reasonably estimated. However, there are some parameters for which no experimental data are known or they may affect the given system significantly. Parameters to be considered are $r, \lambda_{12}, \lambda_G, \mu_{\rho 1},\ \mu_{\rho 2}, \chi_\rho,\ \chi_E,\ \mu_{ED}, \mu_{PM}$. In order to identify how sensitive is the cell populations and

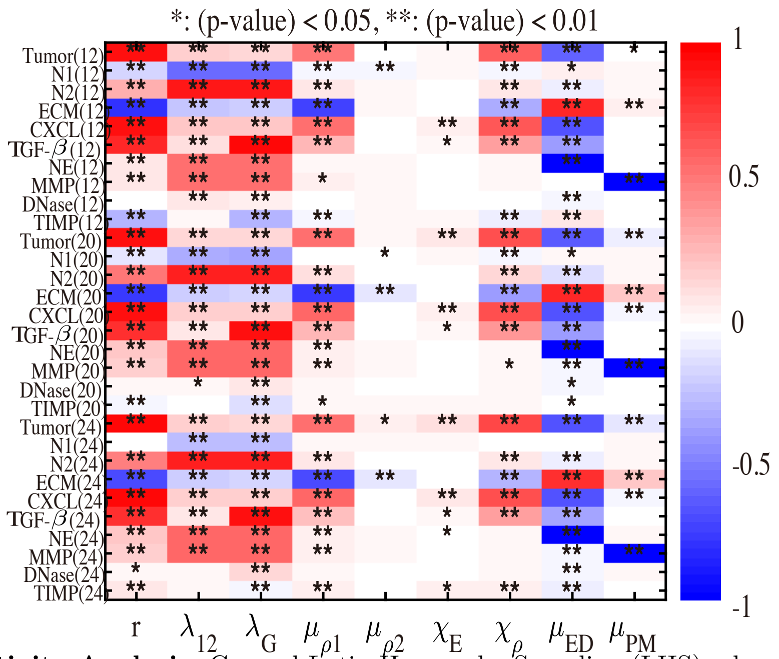

Figure S5. Sensitivity Analysis: General Latin Hypercube Sampling (LHS) scheme and Partial Rank Correlation Coefficient (PRCC) performed on the mathematical model. The reference output is PRCC values (red for positive PRCC values; blue for negative PRCC values) for the populations of cells (tumor cells, N1 TANs, and N2 TANs) and concentrations of molecules (tumor ECM, CXCL, TGF-β, NE, MMPs, DNase, and TIMP) at time $t = 12, 20, 24\ h$.

signaling levels after 12, 20 or 24 *hours* to those chosen parameters, we have performed a sensitivity analysis using a modified version of the general latin hypercube sample (LHS) and partial rank correlation coefficient (PRCC) developed in [14]. The computation was done by simulation results from the PDE model (6)-(18) in the main text with selected parameter sets and the modified Matlab files available from the website of Denise Kirschner's Lab: *http://malthus.micro.med.umich.edu/lab/usadata/*. The analysis was carried out for cell populations and molecule levels from densities of all variables by integration. A physically-reasonable range ($[P_{min}, P_{max}]$) of these parameters was chosen and we divided each range into 5,000 sub-intervals of uniform length while all other parameters were fixed. For those nine parameters of interest, we calculated the PRCC value [14] in the interval $[-1, 1]$. The sign of the PRCC value of a parameter P determines whether an increase in P will increase (+) or decrease (-) the main variable of

interest at a given time ($t = 12, 20, 24\ h$). However, if the absolute value of the PRCC value is small, say close to zero, it doesn't provide a significant correlation between P and the main variable. When the absolute value of the PRCC value is closer to 1, it statistically indicates the strong correlation between P and the main variable.

The computed PRCC values and their associated p-values of all variables for the nine perturbed parameters $(r, \lambda_{12}, \lambda_G, \mu_{\rho 1}, \mu_{\rho 2}, \chi_\rho, \chi_E, \mu_{ED}, \mu_{PM})$ are shown in Fig S5. Here, as before, the population of tumor cells is defined as $\hat{n}(t) = \int_\Omega n(x,t)\ dx$, at t =12 (upper panel), 20 (middle panel), 24 (lower panel) hours. Populations of other cells and levels of molecules are defined in a similar fashion. Based on the PRCC results, we conclude that the tumor cell population is positively correlated to the parameters $r, \mu_{\rho 1}, \chi_\rho$, but is weakly correlated with $\mu_{\rho 2}, \chi_E, \mu_{PM}$. The tumor population is also negatively correlated to the parameter μ_{ED}. While the N1 TAN is insensitive to most parameters, it is negatively correlated with the TAN transition rate (λ_{12}) and TGF-β secretion rate (λ_G). On the other hand, the N2 TAN population is positively correlated with λ_{12} and λ_G, but is only weakly correlated with other parameters. One also observes that the ECM density is negatively correlated with $r, \mu_{\rho 1}$, and χ_ρ, but positively correlated with μ_{ED}. One also can see that the growth rate of tumor cells (r) is very sensitive to the concentrations of CXCL, TGF-β, and ECM while μ_{ED} shows a high sensitivity for tumor cell density and concentration of ECM, CXCL, TGF-β, and NET/NE.

Fig S6 shows the PRCC values of the migratory tumor cell population in the lower chamber for the same perturbed parameters $(r, \lambda_{12}, \lambda_G, \mu_{\rho 1}, \mu_{\rho 2}, \chi_\rho, \chi_E,\ \mu_{ED},\ \mu_{PM})$. Here, the invasive population is defined as $\hat{n}^+(t) = \int_{\Omega_-} n(x,t)\ dx$, at t =12 (blue solid), 20 (red comb), 24 (yellow dot) hours. The numbers of invasive tumor cells are positively correlated with the parameters $r, \mu_{\rho 1}, \chi_\rho$, but not so sensitive to $\lambda_{12}, \lambda_G, \mu_{\rho 2}, \chi_E, \mu_{PM}$. In particular, the invasive capacity of tumor cells will increase significantly if the growth rate of tumor cells (r) or the ECM degradation rate ($\mu_{\rho 1}$) from N2-secreted NE is increased. We note that the migration potential of tumor cell population is also positively correlated with the haptotactic strength χ_ρ. However, the tumor cell invasiveness is negatively correlated with the degradation rate of NE by DNase I (μ_{ED}). Therefore, tumor cell invasion can be sensitively reduced in response to DNase treatment.

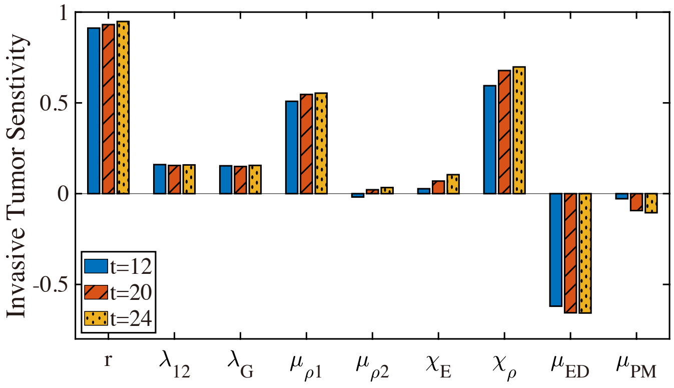

Figure S6. Sensitivity Analysis: PRCC values of migratory tumor cells (cell population in the lower chamber) at time t =12 (blue solid), 20 (red comb), 24 (yellow dot) h.

References

1. Kim Y, Lawler S, Nowicki MO, Chiocca EA, Friedman A. A mathematical model of Brain tumor : pattern formation of glioma cells outside the tumor spheroid core. J Theo Biol. 2009;260:359–371.

2. Park J, Wysocki RW, Amoozgar Z, Maiorino L, Fein MR, Jorns J, et al. Cancer cells induce metastasis-supporting neutrophil extracellular DNA traps. Sci Transl Med. 2016;8(361):361ra138.

3. Kim Y, Friedman A. Interaction of tumor with its microenvironment : A Mathematical Model. Bull Math Biol. 2010;72(5):1029–1068.

4. Welch DR, Schissel DJ, Howrey RP, Aeed PA. Tumor-elicited polymorphonuclear cells, in contrast to normal circulating polymorphonuclear cells, stimulate invasive and metastatic potentials of rat mammary adenocarcinoma cells. Proc Natl Acad Sci U S A. 1989;86(15):5859–63.

5. Manfroi B, Moreaux J, Righini C, Ghiringhelli F, Sturm N, Huard B. Tumor-associated neutrophils correlate with poor prognosis in diffuse large B-cell lymphoma patients. Blood Cancer J. 2018;8(7):66.

6. Hiramatsu S, Tanaka H, Nishimura J, Sakimura C, Tamura T, Toyokawa T, et al. Neutrophils in primary gastric tumors are correlated with neutrophil infiltration in tumor-draining lymph nodes and the systemic inflammatory response. BMC Immunol. 2018;19(1):13.

7. Li Z, Hong N, Robertson M, Wang C, Jiang G. Preoperative red cell distribution width and neutrophil-to-lymphocyte ratio predict survival in patients with epithelial ovarian cancer. Sci Rep. 2017;7:43001.

8. Houghton AM, Rzymkiewicz DM, Ji H, Gregory AD, Egea EE, Metz HE, et al. Neutrophil elastase-mediated degradation of IRS-1 accelerates lung tumor growth. Nat Med. 2010;16(2):219–223.

9. Barker T, Fulde G, Moulton B, Nadauld LD, Rhodes T. An elevated neutrophil-to-lymphocyte ratio associates with weight loss and cachexia in cancer. Sci Rep. 2020;10(1):7535.

10. Chen Y, Yan H, Wang Y, Shi Y, Dai G. Significance of baseline and change in neutrophil-to-lymphocyte ratio in predicting prognosis: a retrospective analysis in advanced pancreatic ductal adenocarcinoma. Sci Rep. 2017;7(1):753.

11. Zeren S, Yaylak F, Ozbay I, Bayhan Z. Relationship Between the Neutrophil to Lymphocyte Ratio and Parathyroid Adenoma Size in Patients With Primary Hyperparathyroidism. Int Surg. 2015;100(7–8):1185–9.

12. Peng B, Wang YH, Liu YM, Ma LX. Prognostic significance of the neutrophil to lymphocyte ratio in patients with non-small cell lung cancer: a systemic review and meta-analysis. Int J Clin Exp Med. 2015;8(3):3098–106.

13. Shimizu K, Okita R, Saisho S, Maeda A, Nojima Y, Nakata M. Preoperative neutrophil/lymphocyte ratio and prognostic nutritional index predict survival in patients with non-small cell lung cancer. World J Surg Oncol. 2015;13:291.

14. Marino S, Hogue IB, Ray CJ, Kirschner DE. A methodology for performing global uncertainty and sensitivity analysis in systems biology. Journal of Theoretical Biology. 2008;254(1):178–196.
